# 23ME-01473, an Fc Effector–Enhanced Anti-ULBP6/2/5 Antibody, Restores NK Cell–Mediated Antitumor Immunity through NKG2D and FcγRIIIa Activation

**DOI:** 10.1158/2767-9764.CRC-24-0478

**Published:** 2025-03-21

**Authors:** Joel S. Benjamin, Abigail Jarret, Shashank Bharill, Pierre Fontanillas, for the 23andMe Research Team, Shruti Yadav, Debasish Sen, Dina Ayupova, Danielle Kellar, Susanne Tilk, Clifford Hom, Zahra Bahrami Dizicheh, I-Ling Chen, Anh N. Diep, Shi Shi, Nives Ivic, Caroline Bonnans, Alex Owyang, Pranidhi Sood, Germaine Fuh, Maike Schmidt, Kimberline Y. Gerrick, Patrick Koenig, Mauro Poggio

**Affiliations:** 1 23andMe, Inc. Therapeutics, South San Francisco, California.; 2 23andMe, Inc. Research, Sunnyvale, California.; 3 Proteros Biostructures GmbH, Planegg, Germany.

## Abstract

The landscape of cancer treatment has been transformed by immune checkpoint inhibitors; however, the failure to benefit a large number of patients with cancer has underlined the need to identify promising targets for more effective interventions. In this study, we leverage 23andMe, Inc.’s large-scale human germline genetic and health database to uncover the previously unknown role of UL16-binding protein 6 (ULBP6), a high-affinity NK group 2D (NKG2D) ligand, in cancer and its promise as an immuno-oncology therapeutic target. We confirm ULBP6 expression in human tumors and demonstrate that soluble ULBP6 shed from tumors circumvents NKG2D activation provided by membrane-anchored NKG2D ligands to inhibit immune cell activation and tumor cell killing. Based on these findings, we developed 23ME-01473, a humanized Fc effector–enhanced antibody that binds to ULBP6 and its closely related family members, ULBP2 and ULBP5. 23ME-01473 effectively blocks soluble ULBP6-mediated immunosuppression to restore the NKG2D axis on NK and T cells to elicit tumor growth control. Moreover, the Fc effector–enhanced design of 23ME-01473 increases its binding affinity to fragment crystallizable gamma receptor IIIa, which, together with 23ME-01473’s binding to membrane-anchored ULBP6/2/5 on cancer cells, allows for augmented antibody-dependent cellular cytotoxicity induction, providing a second activation node for NK cells. Our studies demonstrate the therapeutic potential of an Fc effector–enhanced anti-ULBP6/2/5 antibody to reinvigorate NK cell and T-cell activation and cytotoxicity for the treatment of cancer.

**Significance:**

This study emphasizes the utility of population-based genome-wide assessments for discovering naturally occurring genetic variants associated with lifetime risks for cancer or immune diseases as novel drug targets. We identify ULBP6 as a potential keystone member of the NKG2D pathway, which is important for antitumor immunity. Targeting ULBP6 may hold therapeutic promise for patients with cancer.

## Introduction

Advances in immunotherapy have revolutionized cancer treatment, and among the most successful immunotherapies are immune checkpoint inhibitors (ICI), which aim to reinvigorate T cell–mediated antitumor immunity. However, innate and acquired resistance mechanisms to ICIs, such as the loss of major histocompatibility complex I (MHC I)–mediated antigen presentation on the surface of tumor cells, limit the effectiveness of ICIs in many patients with cancer (1, 2). As such, there is still a high unmet need in oncology to develop novel therapeutics.

Unlike T cells, NK cells recognize and eliminate surface MHC-negative tumor cells independent of MHC priming, making them an attractive immuno-oncology (I/O) target. NK cell responses are regulated by a balance of activating and inhibitory receptors (3). Engagement of an activating receptor, NK group 2D (NKG2D), by membrane-anchored NKG2D ligands (NKG2DL) can trigger proinflammatory cytokine production and cytotoxicity against NKG2DL-expressing cells (3). NK T (NKT) cells and cluster of differentiation 8^+^ (CD8^+^) T cells also express NKG2D constitutively, where NKG2D can act as a costimulatory molecule requiring concomitant or previous T-cell receptor engagement to effectively induce lymphocyte activation (4, 5).

In humans, NKG2D recognizes two families of MHC I–like ligands: MHC class I polypeptide–related sequence A/B (MICA/B) and the human cytomegalovirus (CMV) UL16-binding proteins 1–6 (ULBP1–6; ref. 6). NKG2DL expression is largely absent or low on healthy cells but is induced by cell stressors such as DNA damage, oxidative stress, or viral infection (7, 8). NKG2DLs are upregulated in various cancers: MICA in breast, lung, and ovarian cancers (9), ULBP1–5 in breast cancer (10), and ULBP6/2/5 in lung cancer (11). As NKG2DL expression marks malignant cells for elimination by NKG2D-expressing lymphocytes, NKG2D deficiency leads to the development of more aggressive tumors in mouse models (12).

To evade NKG2D-mediated killing, tumors shed NKG2DLs into an immunosuppressive soluble form that binds NKG2D on NK and CD8^+^ T cells to block the binding of membrane-anchored NKG2DLs to NKG2D or downregulates NKG2D expression, thus impeding NKG2D-mediated activation (13–17). Indeed, elevated soluble NKG2DLs (sNKG2DL) have been detected in the sera of patients with multiple cancers (16, 18, 19). Furthermore, pharmacologic inhibition of MICA/B shedding enhances antitumor immunity and controls tumor growth in animal models (17), suggesting that inhibition of shed NKG2DLs may be a promising mechanism to reinvigorate antitumor immunity.

In this study, we identify ULBP6 as a promising novel I/O drug target based on our I/O genetic signature derived from the 23andMe, Inc.’s germline genetic and health survey database (20). Consistent with previous studies of other NKG2DLs (9–11), ULBP6 is elevated in multiple solid tumors and in the plasma of patients with cancer. Mechanistically, we demonstrate that ULBP6 binds NKG2D with the highest affinity among all NKG2DLs, and its soluble form is sufficient to elicit immunosuppression, even when activating NKG2DLs are present. Based on these findings, we developed 23ME-01473, an antibody that binds ULBP6 to prevent its binding to NKG2D and reverses soluble ULBP6 (sULBP6)–mediated immune suppression. Notably, 23ME-01473 also binds to ULBP2 and ULBP5 due to their high amino acid identity to ULBP6. In addition, the Fc domain of 23ME-01473 is afucosylated, which increases its binding affinity to the fragment crystallizable gamma receptor IIIa (FcγRIIIa), another activating receptor on NK cells, to augment the induction of antibody-dependent cellular cytotoxicity (ADCC) of ULBP6/2/5-expressing tumor cells and serve as a second activating signal for NK cells. Activation of the NKG2D and FcγRIIIa axes by 23ME-01473 provided augmented immune activation and tumor growth control, highlighting the promise of 23ME-01473 to induce antitumor immunity in patients with cancer.

## Materials and Methods

### Overview of study recruitment and data collection

Study participants recruited from the customer base of 23andMe, Inc. provided written informed consent and volunteered to participate in the research online, in accordance with the U.S. Common Rule and under a protocol approved by the external Association for the Accreditation of Human Research Protection Programs–accredited Institutional Review Board, Ethical and Independent Review Services, and the U.S. Department of Health and Human Services (https://www.versiticlinicaltrials.org/salusirb).

Participants were invited to respond to web-based surveys, which collected participants’ medical history. In total, 30 cancer phenotypes and 23 immune phenotypes (online Supplementary Table S1) were analyzed using a genome-wide association study (GWAS) approach.

### Genotyping and variant imputation

DNA extraction and genotyping were performed on saliva samples by Clinical Laboratory Improvement Amendments–certified and College of American Pathologists–accredited clinical laboratories of Laboratory Corporation of America. Samples had a minimum call rate of 98.5%. Samples were genotyped on one of five genotyping platforms and imputed using the UK Biobank (RRID:SCR_012815) 200 K Whole Exome Sequencing and the 23andMe, Inc. reference panels as described previously (21). After quality control processing, the final imputed dataset included 172.8 million variants.

### GWAS

A total of 7.8 million participants of European ancestry were selected using a genetic ancestry classification algorithm (bioRxiv 2014:010512), which included the removal of related individuals (22). The selection of individuals for case–control phenotype analyses was designed to maximize the case sample size by preferentially retaining cases over controls. A principal component analysis of ancestry was computed on 513,000 randomly sampled European participants from the 23andMe, Inc. cohort across all the genotyping platforms as described previously (21).

Association analysis was performed on genotyped and imputed variant dosage data using logistic regression and assuming an additive model of allelic effects. Age, sex, the top six principal components, and dummy variables to account for the genotyping platform were included in the logistic regression as covariables. The association test *P* value was computed using a likelihood ratio test. GWAS summary statistics from both genotyped and imputed data were then combined. When choosing between imputed and genotyped results, the imputed result was reported if either the imputed test passed quality control or a genotyped test was unavailable. Otherwise, the genotyped result was reported. After quality control processing (23), the final GWAS summary statistic dataset included 51.6 million variants.

The genomic boundaries of loci harboring genome-wide significant associations (*P* < 5 × 10^−8^, after genomic inflation correction using linkage disequilibrium score regression; ref. 24) were defined by identifying all variants with *P* < 10^−5^ within the vicinity of a genome-wide significance association and then grouping these regions into intervals with at least 250 kb separation. Within each locus, the 99% credible set was computed using the method of Maller and colleagues (25).

Coding variant annotations were computed using VEP (v99; RRID:SCR_007931; ref. 26) and GENCODE transcripts (v33; RRID:SCR_014966; ref. 27). The association results were annotated with >110 published expression quantitative trait loci (eQTL) and quantitative trait loci (QTL) datasets, including BLUEPRINT (RRID:SCR_003844), DGN, DICE, GEUVADIS (RRID:SCR_000684), GTEx (RRID:SCR_013042), PPMI (RRID:SCR_006431), BLOOD_META, LCL_META, AFR_LCL, BRGR, and INSPIRE.

### mRNA expression analysis

RNA-Seq by Expectation-Maximization normalized expression counts for 32 cancer types (*N* = 9,190) in The Cancer Genome Atlas (RRID:SCR_003193; ref. 28) were downloaded from the UCSC Xena (RRID:SCR_018938) browser database (29) in October 2023 and summarized as the median log_2_ expression values per cancer type. Processed transcript per million values from single-cell RNA sequencing of 18 treatment-naive patients with head and neck squamous cell carcinoma (HNSCC), which included five matched pairs of primary tumors and lymph node metastases, were downloaded from the Gene Expression Omnibus (RRID:SCR_005012) under accession ID GSE103322 (30). Author annotations of cell type (stromal, immune, or malignant cancer cells; *N* = 5,902) were used.

### IHC and immunoassays of human samples

Formalin-fixed, paraffin-embedded tissue and plasma from patients with cancer were purchased from Discovery Life Sciences, BioIVT, and Cureline (RRID:SCR_012546). Five-μm-thick sections were cut from each formalin-fixed, paraffin-embedded block and mounted onto Thermo Fisher Superfrost Plus slides. ULBP6/2/5 IHC staining was performed using a Leica Bond RX autostainer (Leica Biosystems, RRID:SCR_025548). Slides were baked and dewaxed, heated for 20 minutes at 100°C for antigen retrieval, incubated with an anti-ULBP6/2/5 antibody or isotype control (23andMe, Inc.) for 60 minutes at room temperature, detected using a BOND Polymer Refine detection kit (Leica Biosystems), and counterstained with hematoxylin. Slides were then dehydrated in a series of graded alcohol and xylene solutions, coverslipped using a Tissue-Tek Film Automated Coverslipper (Sakura Finetek USA), and imaged with a Pannoramic 250 Flash III scanner (3DHISTECH).

Soluble ULBP6/2/5 was quantified in the plasma of patients with cancer using a Meso Scale Discovery (MSD) sandwich assay. Samples were incubated with biotinylated capture and sulfo-tagged detection anti-ULBP6/2/5 antibodies (23andMe, Inc.) for 2 hours with shaking at 500 revolutions per minute at room temperature, and MSD Gold 96-well streptavidin plates (MSD) were blocked with 1% BSA-TBS (Fisher Scientific and Teknova) buffer for 1 hour at room temperature. After emptying the wells, samples were transferred and incubated in the plates for 1 hour with shaking at room temperature. The plates were washed three times with 1× 10mM Tris, 0.15M Sodium Chloride, 0.05% Tween-20, pH 8.0 buffer (Teknova), incubated with MSD Gold Read Buffer A (MSD), and analyzed with a MESO QuickPlex SQ 120MM Reader (MSD).

### Binding affinity of human NKG2DLs for NKG2D

The binding affinity of human NKG2D for human NKG2DLs was determined by surface plasmon resonance measurements from Biacore 8K or Biacore 8K+ instruments at 37°C. For kinetic measurements, 0.5 μg/mL Fc-tagged human NKG2D was injected into separate flow cells of the Series S Sensor Protein A chip at a flow rate of 30 μL/minute to achieve approximately 25 resonance units. Next, threefold serial dilutions of NKG2DLs in HBS-P buffer (0.01 mol/L HEPES, pH 7.4; 0.15 mol/L NaCl; 0.005% surfactant P20) were injected at the same flow rate. A sensorgram was recorded for each sample and subjected to reference and buffer subtraction prior to analysis using Biacore 8K Evaluation Software. Steady-state analysis was also performed given the fast kinetics (fast on- and off-rates) and the sensorgrams reaching steady state during sample injection. The affinity [equilibrium dissociation constant (K_D_)] was determined by fitting the curve of steady-state binding levels to analyte concentrations.

### Human PBMC isolation and priming

Healthy donor leukopaks (STEMCELL Technologies, Cat# 70500.1) were centrifuged at 300 *g* for 10 minutes at room temperature. After aspirating the supernatant, the cell pellet was resuspended in 25 mL of ammonium–chloride–potassium (ACK) lysis buffer for 7 minutes at room temperature and centrifuged in 25 mL of PBS without Mg^2+^/Ca^2+^ at 300 *g* for 5 minutes at room temperature. Cells were washed in 40 mL PBS and centrifuged as described above twice before being counted using a ViCell cell counter. Cells were centrifuged at 300 *g* for 5 minutes and resuspended in freezing medium (90% FBS and 10% DMSO). Frozen peripheral blood mononuclear cells (PBMC) were thawed in a 37°C water bath, washed in 10 mL PBS without Mg^2+^/Ca^2+^, and centrifuged at 300 *g* for 5 minutes at room temperature prior to use in subsequent *in vitro* experiments.

PBMCs were resuspended in 20 mL of prewarmed X-VIVO 15 medium (Lonza, Cat# 04-418Q) with 20 ng/mL of human recombinant IL-2 (R&D Systems, Cat# 202-IL/CF) and 20 ng/mL of human recombinant IL-15 (R&D Systems, Cat# 247-ILB/CF) for 36 hours to upregulate NKG2D expression on NK and CD8^+^ T cells.

### *In vitro* coculture of PBMCs, COV644, and NKG2DLs

A total of 120,000 IL-2–/IL-15–primed PBMCs/well were cocultured with 50,000 COV644 cells/well (Sigma Aldrich, Cat# 07071908-1VL, RRID: CVCL_2425**)**, either recombinant ULBP6-02 (23andMe, Inc.) or MICA (R&D Systems, Cat# 1300-MA-050), and anti-ULBP6/2/5, isotype, or no antibodies in RPMI medium + 10% FBS in a 96-well flat-bottom plate at 37°C. Supernatants were harvested after 24 hours for IFNγ concentration determination using the Luminex ProcartaPlex simplex kit (Thermo Fisher Scientific, Cat# EPX01A-10228-901). Cells were harvested after 48 hours for flow cytometric analysis. PBMCs were stained with Human TruStain FcX (BioLegend, Cat# 422302, RRID:AB_2818986) and LIVE/DEAD Fixable Blue Dead Cell Stain Kit (Thermo Fisher Scientific, Cat# L34962) in PBS for 15 minutes at room temperature, washed with FACS buffer (2% FBS in PBS), and stained with anti-CD4 BUV661 (1:200; BD, Cat# 612962, RRID:AB_2870238), anti-CD8 BUV496 (1:200 dilution; BD, Cat# 612942, RRID:AB_2870223), anti-CD56 BV421 (1:200 dilution; BD, Cat# 562751, RRID:AB_2732054), anti-CD3 BV605 (1:200 dilution; BD, Cat# 563219, RRID:AB_2738076), and anti-CD314 (NKG2D) APC (1:200 dilution; BioLegend, Cat# 320808, RRID:AB_492962) in Brilliant Stain Buffer Plus (BD, Cat# 566385) for 30 minutes at 4°C. Cells were washed with FACS buffer, fixed with eBioscience IC Fixation Buffer (Thermo Fisher Scientific, Cat# 00-8222-49) for 20 minutes at 4°C, and permeabilized (Thermo Fisher Scientific, Cat# 88-8824-00) prior to staining with anti-Ki-67 FITC (1:200; BioLegend, Cat# 151204, RRID:AB_2566800) and anti-granzyme B PerCP/Cyanine5.5 (1:200; BioLegend, Cat# 372212, RRID:AB_2728379) in Perm buffer (BD, Cat# 554723) for 30 minutes at 4°C. Cells were then washed, resuspended in FACS buffer, and analyzed using a Beckman Coulter’s CytoFLEX LX system (RRID:SCR_025067).

PBMCs were also cultured with plate-bound NKG2DLs. Briefly, 96-well flat-bottom plates were coated with 200 nmol/L ULBP4 (R&D Systems, Cat# 6285-UL-050) or MICA (R&D Systems, Cat# 1300-MA-050) in PBS without Mg^2+^/Ca^2+^ overnight. The next day, the plates were washed twice with PBS, blocked with 2% BSA in PBS for 30 minutes, and incubated with 100,000 IL-2–/IL-15–primed PBMCs with or without recombinant ULBP6-02 (23andMe, Inc.) for 24 hours at 37°C. Supernatants were collected and analyzed for IFNγ as described previously.

COV644 cells cultured alone were trypsinized, stained with LIVE/DEAD Fixable Blue Dead Cell Stain, and blocked with Human TruStain FcX (1:100; BioLegend, Cat# 422302, RRID:AB_2818986) in FACS buffer for 15 minutes at 4°C. Cells were stained with anti-MICA/B (1:20; BioLegend, Cat# 320906, RRID:AB_493193), anti-ULBP6/2/5 (1:20; R&D Systems, Cat# FAB1298P, RRID:AB_2214693) antibodies, or an isotype control in FACS buffer containing Brilliant Buffer, washed, and analyzed as described above.

### COV644-GFP and MC38-ULBP6 cell line generation

COV644 stably expressing GFP (COV644-GFP) was transduced with a CMV lentiviral construct for 2 weeks before being sorted with a Sony SH800S cell sorter system (RRID:SCR_018066). COV644-GFP cells were used for *in vitro* tumor cell killing assays.

ULBP6-02 encoding plasmids were generated by site-directed mutagenesis of ULBP6-01 sequences amplified from COV644 cDNA and inserted into a pCDH-CMV-MCS2 cloning and expression lentivector (System Biosciences, Cat# CD501A-1) with amino acid changes at G26R, R106L, and I147T. MC38 cell lines (Kerafast, Cat# ENH204-FP, RRID:CVCL_B288) overexpressing ULBP6-02 were generated using the Lenti-X Lentiviral (Takara Bio) expression system. Briefly, ULBP6-02–encoding or empty vector plasmids were transfected into Lenti-X 293T cells using Lipofectamine LTX Reagent (Thermo Fisher Scientific, Cat# 15338100). Forty-eight hours later, viral supernatant was filtered through a 0.45-μm filter (Corning, Cat# 431220) and concentrated using Lenti-X Concentrator (Clontech, Cat# 631231). MC38 cells were incubated with the supernatant overnight, and the cell culture medium was refreshed for 24 hours prior to antibiotic selection. MC38-transfected cells were stained with anti-ULBP6 (R&D Systems, Cat# FAB1298A, RRID:AB_2257142) to confirm ULBP6 expression by flow cytometry.

Cell line authentication was not performed for the COV644 or MC38 cell lines. Mycoplasma testing was performed on both cell lines using PCR by IDEXX BioAnalytics twice a month throughout the duration of our studies. Cells used for the described experiments did not exceed 15 to 20 passages.

### *In vitro* tumor cell killing assays

A total of 3,000 COV644-GFP cells were seeded per well of a 96-well PerkinElmer CellCarrier Ultra plate for 16 hours before the addition of recombinant ULBP6-02, antibodies, and 75,000 IL-2/IL-15–primed PBMCs/well, 45,000 NK cells purified from primed PBMCs using the EasySep Human NK Cell Isolation Kit (STEMCELL Technologies, Cat# 17955), or 45,000 IL-2–primed (primed with 20 ng/mL IL-2 for 36 hours) T cells purified using Dynabeads CD3 (Thermo Fisher Scientific, Cat# 11151D). The cocultures were incubated at 37°C in an Incucyte S3 live cell imager (Sartorius, RRID: SCR_023147) for 4 to 5 days, and images were taken every 4 hours. The area of GFP expression per well per time point was calculated and normalized to time 0.

### *In vivo* studies of ULBP6-overexpressing MC38 model

All animal studies and procedures were performed in accordance with the approved protocols of the Institutional Animal Care and Use Committee of 23andMe, Inc. and the regulations of the Association for Assessment and Accreditation of Laboratory Animal Care International. ULBP6-02– or empty vector–overexpressing MC38 cells were injected into the right rear flank of 10-week-old female B cell–deficient μMT^−/−^ mice (The Jackson Laboratory, B6.129S2-Ighmtm1Cgn/J, Cat# 002288, RRID: IMSR_JAX:002288). Nineteen days after inoculation, tumors were harvested and dissociated using the gentleMACS Dissociator (Miltenyi Biotec, Cat# 130-0960427), filtered through a 70 μmol/L filter, and incubated with ACK buffer for red blood cell lysis for flow cytometric analysis. Cells were processed as described in a previous section with the following changes. Cells were blocked with FcR Blocking Reagent (Miltenyi Biotec, Cat# 130-092-575) in FACS buffer for 15 minutes at 4°C prior to staining with anti-NK1.1 BV605 (1:100; BioLegend, Cat# 108740, RRID:AB_2562274), anti-CD8 BUV496 (1:200; BD, Cat# 750024, RRID:AB_2874242), anti-CD45 BUV661 (1:200; BD, Cat# 612975, RRID:AB_2870247), anti-CD27 BV421 (1:100; BD, Cat# 740028, RRID:AB_2739800), anti-CD11b BV650 (1:100; BD, Cat# 563402, RRID:AB_2738184), anti-CD90.2 APC-Cy7 (1:100; BD, Cat# 561641, RRID:AB_10898013), anti-CD25 BV785 (1:100; BD, Cat# 564368, RRID:AB_2738771), anti-CD69 AF700 (1:100; BioLegend, Cat# 104539, RRID:AB_2566304), anti-PD-1 BV510 (1:100; BioLegend, Cat# 135241, RRID:AB_2715761), and anti-NKG2D APC (1:100; BioLegend, Cat# 130212, RRID:AB_1236372) in Brilliant Stain Buffer (BD, Cat# 563794). After intracellular fixation and permeabilization, cells were stained with anti-Ki-67 PE (1:100; BD, Cat# 567719, RRID:AB_2916708), anti-granzyme B FITC (1:100; BioLegend, Cat# 515403, RRID:AB_2114575), and anti-IFNγ PerCP-Cy5.5 (1:100; BioLegend, Cat# 505822, RRID:AB_961359) antibodies overnight at 4°C.

Sera were also harvested for soluble ULBP6/2/5 quantification using a ULBP-2 DuoSet ELISA (R&D Systems, Cat# DY1298). Briefly, 96-well plates were coated with the capture antibody overnight at room temperature. The next day, the plates were washed three times, blocked for 1 hour with PBS supplemented with 1% BSA, washed again, and incubated with sera for 2 hours at room temperature. The plates were then washed, incubated with the detection antibody diluted for 2 hours at room temperature, washed again, and detected with streptavidin–horseradish peroxidase followed by tetramethylbenzidine substrate. After 20-minute incubation, stop solution was added, and the OD_450_ of the plates was read on a SpectraMax i3 instrument.

### Anti-ULBP6/2/5 antibody generation

BALB/c, Swiss Webster, NZB/W, and SJL mice were immunized with human ULBP6-01 (residues G26-G218) and ULBP6-02 extracellular domain (residues R26-G218) using the Sigma adjuvant system (Millipore Sigma); Freund’s Adjuvant, Complete (Millipore Sigma); TiterMax Gold Adjuvant (Millipore Sigma); GERBU Adjuvant (V-Biognostics); and TLR cocktail (InvivoGen) as adjuvants. Mice with sufficient antigen-specific serum titers received a final boost without adjuvant, and fusion was performed 3 days later. Hybridomas were generated by electrofusioning splenocytes and lymphocytes with the SP2/mIL-6 mouse myeloma cell line (ATCC, Cat# CRL-2016, RRID: CVCL_C561) and selected using ClonaCell-HY Hybridoma buffers (STEMCELL Technologies). The supernatants of hybridoma clones producing anti-ULBP6 antibodies were confirmed to bind human ULBP6-01, ULBP6-02, and ULBP2 using an ELISA and assessed for binding to ULBP proteins on CHO-S cells overexpressing human ULBP1, ULBP2, ULBP3, ULBP5, ULBP6-01, or ULBP6-02 via flow cytometry (Sartorius). The heavy- and light-chain variable domain sequences of hybridoma clones were determined by an Illumina MiSeq System. Fc-WT-anti-ULBP6/2/5 antibodies were selected for humanization using a classical complementarity-determining region grafting approach to the closest human framework while maintaining selected framework positions from the parental sequence, which are known to be important for maintaining binding and activity (31). DNA constructs encoding the heavy or light chain of Fab proteins and full-length antibodies were synthesized and cloned (GENEWIZ/Azenta). DNA constructs for Fab proteins were transfected into ExpiCHO-S cells (Thermo Fisher Scientific, Cat# A29127, RRID: CVCL_5J31) using a 1:1 ratio of heavy chain to light chain, and the recombinant Fabs were purified from the supernatant using HisPur Ni-NTA resin (Thermo Fisher Scientific) and buffer-exchanged into phosphate-buffered saline (PBS) using a PD10 desalting column (GE Healthcare). DNA constructs for full-length antibodies were transfected into either ExpiCHO-S cells for Fc-attenuated (N297G) or CHO FUT8-KO for Fc-enhanced afucosylated versions using a 1:2 ratio of heavy chain to light chain. The recombinant antibodies were purified from the supernatant using Protein A affinity chromatography and size exclusion chromatography with a Superdex 200 column (Cytiva). Potential off-target binding of the humanized antibodies was assessed using baculovirus ELISA (32).

### 23ME-01473 characterization

23ME-01473 was selected as the lead anti-UBP6/2/5 antibody based on its specificity for human ULBP6, affinity for human and cyno ULBP6/2, capacity to block the binding of human ULBP6/2/5 to human NKG2D (as described below), activity in *in vitro* cell-based assays, and temperature- and oxidation-stressed developability assessment assays.

The binding affinity of 23ME-01473 for human ULBPs was determined by surface plasmon resonance measurements, as described in the previous section. Association rates (k_a_) and dissociation rates (k_d_) were calculated using a one-to-one Langmuir binding model. K_D_ was calculated as the ratio of the dissociation and association rates (k_d_/k_a_).

The binding affinities of 23ME-01473 and Fc-WT-anti-ULBP6/2/5 antibodies for human Fcγ receptors were determined by surface plasmon resonance measurements as described in a previous section (binding affinity of human NKG2DLs for NKG2D) with the following modifications. Biotinylated FcγRs were injected into separate flow cells of the Series S Sensor Chip CAP, and threefold serial dilutions of the anti-ULBP6/2/5 antibodies were injected at the same flow rate.

The capacity of 23ME-01473 to block the binding of ULBP2, ULBP5, and ULBP6-02 to NKG2D was evaluated using ELISAs. Briefly, 96-well MaxiSorp flat-bottom plates were coated with Fc-tagged NKG2D and subsequently blocked with PBS supplemented with 1% BSA and 0.05% Tween 20. After washing with 0.05% Tween 20 in PBS, threefold serial dilutions of biotinylated ULBPs were added to the MaxiSorp plates. The plates were then washed, incubated with poly-horseradish peroxidase–labeled streptavidin, and developed with colorimetric tetramethylbenzidine substrate and 2N H_2_SO_4_. The optical density at 450 nm (OD_450_) was read on a SpectraMax i3x plate reader, and the EC_80_ was determined using GraphPad Prism software (RRID:SCR_002798).

For the blocking assay, 96-well MaxiSorp flat-bottom plates were coated overnight with Fc-tagged NKG2D, blocked with PBS supplemented with 1% BSA and 0.05% Tween 20, and washed with 0.05% Tween 20 in PBS. The corresponding EC_80_ for ULBP5 (1.55 nmol/L) and ULBP6-02 (0.15 nmol/L) was precomplexed with 23ME-01473 and transferred to the plates. The plates were then washed, developed, and analyzed as described in the previous section. IC_50_ values were determined by fitting the measured OD_450_ and log_10_23ME-01473 concentration to a dose–response curve using the GraphPad Prism software and the following equation: Y = Bottom + (Top − Bottom)/(1 + 10^[logIC^^50^^−logX] × HillSlope^).

### Crystallization of 23ME-01473 Fab in complex with ULBP6

ULBP6-02 (residues 26-218) and 23ME-01473 Fab were complexed at a ratio of 1.2:1 and purified via size exclusion chromatography using a HiLoad 16/60 Superdex 75 prep grade column preequilibrated in 20 mmol/L HEPES, pH 7.0, and 100 mmol/L NaCl. 23ME-01473 Fab and ULBP6-02 cocrystals were grown by sitting drop vapor diffusion at 4°C by mixing equal volumes of 0.1 μL protein complex (26.7 mg/mL) with 0.1 μL of reservoir solution containing 10% glycerol (v/v), 0.1 M MES (pH 6.9), 5% PEG 1000 (w/v), and 30% PEG 600 (w/v). Rod-shaped crystals were observed after 3 to 5 days and flash-frozen in liquid nitrogen. Diffraction data collection was performed at 100 K and 0.9998Å wavelength using a beamline PXII-X10SA and a Dectris EIGER2 S 16M detector and processed using the autoPROC (RRID:SCR_015748), X-ray Detector Software (RRID:SCR_015652), and AIMLESS software (RRID:SCR_015747; refs. 33–38) at the Swiss Light Source. Phase information was obtained by molecular replacement using Phaser (RRID:SCR_014219) from the CCP4 (39) software package (RRID:SCR_007255) and ULBP6 [Research Collaboratory for Structural Bioinformatics Protein Data Bank (RCSB PDB), RRID:SCR_012820, entry 4S0U] as the template. Subsequent model building and refinement were performed according to standard protocols with COOT (RRID:SCR_014222; ref. 40) and the software package CCP4, respectively. Model quality was evaluated with MolProbity (RRID:SCR_014226; ref. 41). The asymmetric unit contains one molecule of ULBP6-02 and one heavy chain and light chain of 23ME-01473 Fab. The following structures were fully resolved: the heavy-chain Ca backbone from residues 2 to 232 (Kabat numbering; 219, Eu numbering), the light chain from residues 1 (Kabat numbering) to 213 (Kabat and Eu numbering), and ULBP6-02 from residues 29 to 194 (Uniprot, RRID:SCR_002380, numbering as in entry Q5VY80). This work was performed by Proteros and is deposited in RCSB PDB (entry ID 8RWB).

### NKG2D and FcγRIIIa combinatory activity

Ninety-six-well flat-bottom plates were coated overnight with Fc-Att-anti-lysozyme antibody that carries an N297G mutation in the Fc domain (X-lys-E−), Fc-WT-anti-lysozyme antibody (X-lys-E+), Fc-Att-anti-lysozyme antibody that carries an N297G mutation in the Fc domain and has ULBP6 fused to the light chain (ULBP6-lys-E−), or Fc-WT-anti-lysozyme antibody that has ULBP6 fused to the light chain (ULBP6-lys-E+). The next day, the plates were washed with PBS without Mg^2+^/Ca^2+^, blocked with PBS supplemented with 2% BSA for 20 minutes, washed again, and incubated with 200,000 IL-2/IL-15–primed PBMCs at 37°C for 24 hours. Following the incubation, the supernatant was collected and analyzed using an IFNγ ELISA (Invitrogen, Cat# 88-7316-88) or ProcartaPlex Luminex IFNγ (Thermo Fisher Scientific, Cat# EPX01A-10228-901) per the manufacturer’s instructions.

### ADCC reporter assay

A total of 25,000 COV644 cells/well were plated in a 96-well white plate overnight in a tissue culture incubator with 5% CO_2_ at 37°C. The next day, anti-ULBP6/2/5 or isotype antibodies were added, and the plates were used with the Promega ADCC Reporter Bioassay (Promega, Cat# G7010) per the manufacturer’s instructions. The relative light units from the background wells, which contained only Bright-Glo Luciferase Assay Reagent, were subtracted from all the wells.

### *In vivo* studies of a PDX model

All experimental procedures were performed according to Champions Oncology’s Institutional Animal Care and Use Committee guidelines. Champions TumorGraft model CTG-3470 non–small cell lung cancer (NSCLC) patient-derived xenograft (PDX) tumors were implanted subcutaneously in the left flank of hIL-15 NOG female mice (Taconic Inc.) and harvested for flow cytometry, IHC, and ELISA. When the tumor volume reached approximately a median of 150 mm^3^, animals were matched by tumor size and assigned to treatment or control groups (*N* = 18 per group). NK cells were isolated from healthy donor PBMCs (*N* = 3 biological replicates) and expanded *ex vivo*. Cells (10 × 10^6^) were intravenously transplanted within 1 day after randomization (day 0; *N* = 6 mice per donor PBMC) to maintain NK cell persistence throughout the study duration (42). NK cell viability was assessed and documented at the time of preparation for transplantation. Four hours after NK cell engraftment (day 0), animals were dosed twice a week with 10 mg/kg 23ME-01473 in the treated group or afucosylated hIgG1 isotype control in the control group for 6 weeks (a total of 12 doses). Tumor growth was monitored twice a week using digital calipers, and the tumor volume was calculated using the following formula: 0.52 × (length × width^2^). Tumor size and body weight were measured twice weekly for 58 days following randomization.

Tumor growth inhibition was determined using the following equation: $100\%\;\;\times \;1-\;\frac{\mathrm{final}\;\mathrm{mean}\;\mathrm{tumor}\;\mathrm{volume}\;\left(\mathrm{MTV}\right)-\mathrm{initial}\;\mathrm{MTV}\;\mathrm{of}23\mathrm{ME}-01473\;\mathrm{group}}{\mathrm{final}\;\mathrm{MTV}-\mathrm{initial}\;\mathrm{MTV}\;\mathrm{of}\;\mathrm{isotype}\;\mathrm{control}\;\mathrm{group}}$. Statistical analysis was done using an adjusted area under the curve (R software), which was calculated for each mouse to quantitatively assess tumor volume over time. The analysis centered on the ratio of the mean adjusted areas under the curve between the control and treated groups. Confidence intervals for each ratio were calculated using a nonparametric bootstrapped t-interval method, as previously described (43). A one-sided test was conducted to ascertain if each ratio was significantly less than 1, suggesting lower tumor volume in the treated group. The statistical significance of this finding was evaluated using an FDR-adjusted alpha level of 0.05, which provides stringent control of the type I error rate in the context of multiple testing scenarios.

PDX tumors collected from animals were analyzed for flow cytometry, IHC, and ELISA. For flow cytometric analysis, PDX tumors were processed as described previously with the following changes. Cells were blocked with TruStain FcX (anti-mouse CD16/32) antibody (1:100; BioLegend, Cat# 101319, RRID:AB_1574973), whole human IgG (200 μg/mL; Sigma, Cat# I4506-50MG, RRID:AB_1163606), and 10 mmol/L EDTA in FACS buffer for 15 minutes at 4°C. Cells were stained with anti-CD45 FITC (1:20; BioLegend, Cat# 103108, RRID:AB_312973), anti-EpCAM APC-Cy7 (1:20; BioLegend, Cat# 324246, RRID:AB_2783194), anti-ULBP6/2/5 PE (1:10; R&D Systems, Cat# FAB1298P, RRID:AB_2214693), and anti-MICA/B APC (1:10; R&D Systems, Cat# FAB13001A, RRID:AB_663946) or isotype controls mIgG2a PE (1:10; R&D Systems, Cat# IC003P, RRID:AB_357245) or mIgG2a APC (1:10; R&D Systems, Cat# IC003A, RRID:AB_357243). Tumors were formalin-fixed, paraffin-embedded and stained with anti-MICA/B (Abcam, Cat# ab203679) or anti-ULBP6/2/5 (23andMe, Inc.) for IHC at Acepix Biosciences. For ULBP6/2/5 ELISA, tumors were digested in T-PER Tissue Protein Extraction Buffer (Thermo Fisher Scientific, Cat# 78510) with Halt protease inhibitor cocktail (1:100; Thermo Fisher Scientific, Cat# 87785) using a Miltenyi gentleMACS dissociator. Cell suspensions were centrifuged at 10,000 *g* for 5 minutes at 4°C, and the clarified supernatants were transferred to a new tube and frozen at –80°C. Quantification of ULBP6/2/5 was done using an anti-ULBP6/2/5 ELISA as described previously.

### Exosome isolation and immunoblotting

COV644 cell pellets and supernatants were harvested after 48 hours of culturing. Cell pellets were washed twice with PBS without Mg^2+^/Ca^2+^, incubated with RIPA buffer supplemented with a protease inhibitor cocktail for 15 minutes at 4°C, and centrifuged at 14,000 rpm for 15 minutes at 4°C.

Exosomes were isolated from the supernatants by centrifugation at 300 *g* for 5 minutes at room temperature, followed by 2,000 *g* for 20 minutes at 4°C and then 10,000 *g* for 30 minutes at 4°C. The supernatant was gently layered onto a 30% w/v sucrose solution in an ultracentrifuge tube and centrifuged at 100,000 *g* for 90 minutes at 4°C. The pelleted exosomes were resuspended in PBS without Mg^2+^/Ca^2+^, centrifuged at 100,000 *g* for 70 minutes at 4°C, and resuspended in RIPA buffer for immunoblotting analysis.

Total protein concentration from cell lysates and isolated exosomes was quantified by a Pierce BCA protein assay (Thermo Fisher Scientific, Cat# 23225). Ten micrograms of total protein per sample was loaded onto 4% to 20% Mini-PROTEAN TGX precast gels (Bio-Rad, Cat# 4561093) and transferred onto nitrocellulose membranes. The blots were blocked with Intercept (PBS) Blocking Buffer (LI-COR, Cat# 927-90001); incubated with anti-ULBP6/2/5 (1:2,000, Thermo Fisher Scientific, Cat# PA5-47118, RRID:AB_2607294), anti-CD63 (1:1,000, Abcam, Cat# ab271286), and anti-CD9 (1:1,000, Abcam, Cat# ab236630, RRID:AB_2922400) overnight at 4°C; detected with IRDye secondary antibodies (LI-COR), and developed using a LI-COR Odyssey CLx imager.

### Statistical analysis

All statistical analyses, excluding the analysis of the PDX tumor growth inhibition, were performed in GraphPad Prism, with comparisons between two groups using parametric *t* tests and comparisons with more than two groups using one-way ANOVA with multiple comparisons (ordinary one-way ANOVA). Where applicable, EC_50_ curves were calculated with a nonlinear regression model with a log(agonist) versus response variable slope (four parameters).

### Data availability

The crystal structure of ULBP6 in complex with NKG2D and the Fc-WT anti-ULBP6/2/5 antibody is deposited in the RCSB PDB (entry IDs 4S0U and 8RWB, respectively). All other data generated in this study are available within the article and its supplementary data files, except for 23andMe, Inc.’s genetic and phenotypic database, which cannot be shared to maintain the privacy of our participants.

## Results

### Identification of ULBP6 (*RAET1L*) as a novel I/O target

**Figure 1:**
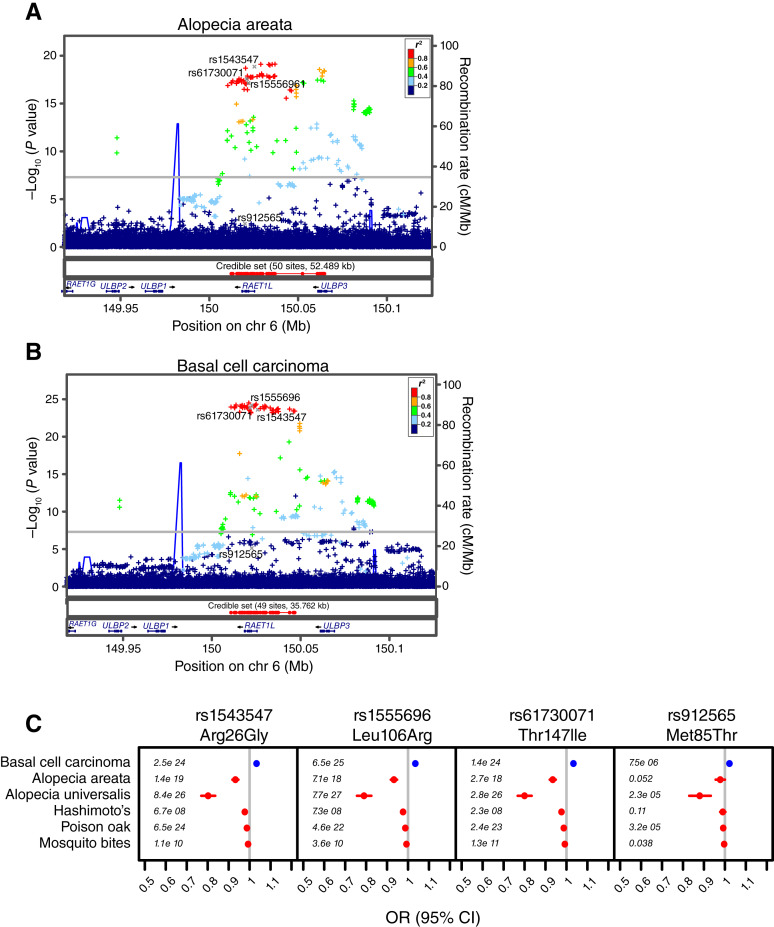
*RAET1L* (ULBP6) germline genetics. Regional GWAS plots of the three germline *RAET1L* variants: rs1543547, rs1555696, and rs61730071, identified in the credible set that are significantly associated with (**A**) alopecia areata and (**B**) basal cell carcinoma. The fourth germline *RAET1L* variant, rs912565, is not significantly associated with either phenotype. **C,** The three *RAET1L* variants, but not rs912565, showed significant (*P* < 5 × 10^−8^) associations, as measured by ORs and *P* values (annotated in italics), with basal cell carcinoma (shown in blue) and immunologic diseases (shown in red).

Given the opposing immunologic etiology of cancer versus autoimmunity and inflammation, we hypothesized that genetic loci exhibiting inverse risk for cancer and immunologic diseases may be important regulators of host immunity and thus promising drug targets for cancer (2, 3). As such, we developed an I/O genetic signature (20) that identifies naturally occurring genetic loci in the human population with significant (*P* value < 5 × 10^−8^) genome-wide opposing associations with cancer and immune-related diseases using genome- and phenome-wide association studies of 23andMe, Inc.’s germline genetic and health database, comprising data from 7.8 million consenting participants of European descent (Supplementary Table S1). One of the loci identified is located on chromosome 6, in which five *ULBP* genes are clustered. Significant GWAS signals were observed in alopecia areata (Fig. 1A), basal cell carcinoma (Fig. 1B), alopecia universalis, Hashimoto’s disease, poison oak, and mosquito bites (Supplementary Table S2). The credible set was composed of 36 variants, which were most significantly associated with alopecia universalis and overlapped with the *RAET1L* gene (which encodes ULBP6). Of the four *RAET1L* germline missense variants found in the coding region of the gene that do not result in the loss of ULBP6 expression and were used to define four previously reported ULBP6 isoforms (44), three [rs1543547 (T/C, Arg26Gly), rs1555696 (A/C, Leu106Arg), and rs61730071 (G/A, Thr147Ile)] were identified in this credible set. The fourth germline missense *RAET1L* variant, rs912565 (A/G, Met85Thr), was not significantly associated with any cancer or immune-related diseases (Fig. 1C; Supplementary Table S2). Although not statistically significant, other cancer phenotypes, such as squamous cell carcinoma, melanoma, and hematologic malignancies, exhibited a similar directionality to basal cell carcinoma with the four *RAET1L* missense variants (Supplementary Fig. S1).

The presence of the three *RAET1L* variants in the credible set suggests *RAET1L* as the probable causal gene in this locus. We further annotated the GWAS signal using >110 published eQTL and QTL datasets. The observed association signal harbored eQTL polymorphisms significantly regulating the expression of four of the five *ULBP* genes in this cluster (*RAET1G* as the exception) in primarily skin and mucosal tissues (Supplementary Table S3). This suggests that the variants present in the associated signals may alter not only *RAET1L* expression but also *ULBP1*, *ULBP2*, and *ULBP3* and affect the risk of cancer and immune diseases.

Taken together, the overlap of the GWAS signal from the credible set over *RAET1L* and the association of coding variants and eQTLs suggest that the likely causal gene is *RAET1L*. Although *RAET1L* is associated with inverse risks for cancer and inflammatory diseases, the directionality of its expression based on our genetics data alone in either disease class is unclear. Thus, we subsequently characterized the expression and function of *RAET1L* (ULBP6) in disease models.

### ULBP6 is expressed in human cancers

**Figure 2:**
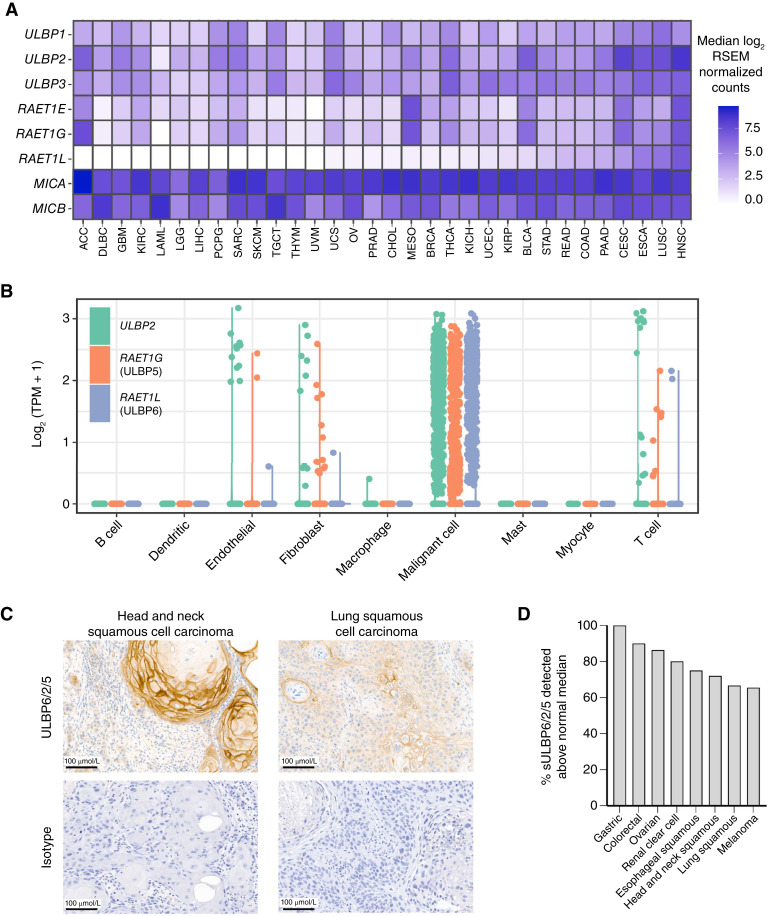
mRNA and protein expression of NKG2D ligands in human cancers. **A,** Heatmap of the median mRNA expression of NKG2DLs per cancer type in The Cancer Genome Atlas. Cancer types are sorted by median expression of *RAET1L* (ULBP6). **B,** mRNA expression of *ULBP2*, *RAET1G*, and *RAET1L* (ULBP5) in stromal, immune, and malignant cancer cells (*N* = 5,902) from 18 treatment-naive patients with HNSC. **C,** Representative IHC images of ULBP6/2/5 expression in HNSC and LUSC human tumors (top) and the corresponding isotype controls (bottom). **D,** sULBP6/2/5 levels in the plasma of patients with cancer are depicted as the percentage of samples with levels above the median of healthy individuals. ACC, adrenocortical carcinoma; BLCA, bladder urothelial carcinoma; BRCA, breast invasive carcinoma; CESC, cervical squamous cell carcinoma and endocervical adenocarcinoma; CHOL, cholangiocarcinoma; COAD, colon adenocarcinoma; DLBC, lymphoid neoplasm diffuse large B-cell lymphoma; ESCA, esophageal carcinoma; GBM, glioblastoma multiforme; KICH, kidney chromophobe; KIRC, kidney renal clear cell carcinoma; KIRP, kidney renal papillary cell carcinoma; LAML, acute myeloid leukemia; LGG, brain lower grade glioma; LIHC, liver hepatocellular carcinoma; LUSC, lung squamous cell carcinoma; MESO, mesothelioma; OV, ovarian serous cystadenocarcinoma; PAAD, pancreatic adenocarcinoma; PCPG, pheochromocytoma and paraganglioma; PRAD, prostate adenocarcinoma; READ, rectum adenocarcinoma; SARC, sarcoma; SKCM, skin cutaneous melanoma; STAD, stomach adenocarcinoma; TGCT, testicular germ cell tumors; THCA, thyroid carcinoma; THYM, thymoma; UCEC, uterine corpus endometrial carcinoma; UCS, uterine carcinosarcoma; UVM, uveal melanoma.

Given the genetic association of *RAET1L* with cancer, we analyzed its mRNA expression using The Cancer Genome Atlas to confirm its relevance in a broad range of cancers (28). *RAET1L* is highly expressed in head and neck, esophageal, cervical, and lung squamous cell carcinomas (Fig. 2A). In contrast, other NKG2DLs, such as *MICA/B*, exhibited high expression across most of the evaluated cancers. Furthermore, *RAET1L* was expressed primarily in malignant cells, but not in stromal or immune cells (Fig. 2B) within a head and neck squamous carcinoma tumor microenvironment (30), which is consistent with previous findings (45). *ULBP2* and *RAET1G* (which encodes ULBP5), which share the highest sequence homology with *RAET1L*, also exhibit similar expression profiles within the tumor microenvironment. In comparison, *MICA/B* was broadly and highly expressed in both malignant and normal stromal and T cells (Supplementary Fig. S2). The more selective expression of *RAET1L* in malignant cells suggests that ULBP6 may be a promising therapeutic target with a potentially larger therapeutic window.

We next confirmed ULBP6 protein expression across diverse cancer types. Notably, ULBP6 antibodies also recognized ULBP2 and ULBP5, given their high sequence homology (44). Consistent with the mRNA data, lung squamous ecll carcinoma and HNSCC tumors exhibited high expression of ULBP6/2/5, with variable patterns of membranous and nonmembranous ULBP6/2/5 indicative of a differential degree of ULBP6/2/5 shedding (Fig. 2C). Given the detectability of shed NKG2DLs from tumor cells in circulation (16, 18, 19, 46), we assessed the presence of sULBP6/2/5 in a selection of plasma of patients with cancer. Plasma from all evaluated patients with cancer had detectable sULBP6/2/5, and at least 65% of patients with a given cancer type had sULBP6/2/5 levels greater than the median levels measured in healthy donors (Fig. 2D). The presence of elevated ULBP6/2/5 on malignant tumor cells and in the plasma of patients with cancer suggests that ULBP6/2/5 may be involved in regulating the immune response to certain cancers. Indeed, previous reports have demonstrated that elevated sULBP2 in the sera of patients with cancer is associated with poor disease diagnosis and survival (16, 18, 47).

### ULBP6 exhibits the highest affinity for NKG2D among all NKG2DLs

**Figure 3:**
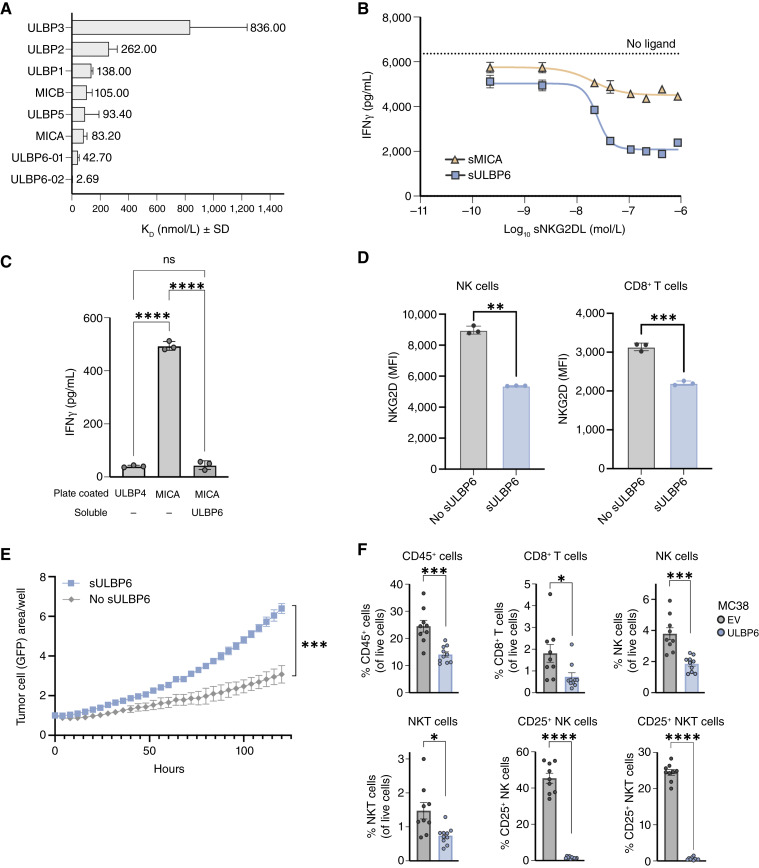
ULBP6 may be the most potent NKG2D ligand. **A,** Equilibrium dissociation constant K_D_ for human NKG2DLs binding to NKG2D as measured by a Biacore surface plasmon resonance assay. Data represent mean ± SD of three technical replicates per condition. ULBP4 is not shown, as no binding to NKG2D was detected. **B,** IFNγ concentration of the supernatants of IL-2/IL-15–primed PBMCs cocultured with COV644 cells and 0.05 to 200 nmol/L recombinant sULBP6-02 or sMICA for 24 hours, depicted as a concentration-dependent response. No IFNγ was detected in COV644 cell supernatants without PBMC. Data represent mean ± SEM of three technical replicates per condition from one of three biological replicates. **C,** IFNγ concentration of the supernatants of IL-2/IL-15–primed PBMCs cocultured with 200 nmol/L plate-bound ULBP4 or MICA ± 250 nmol/L recombinant sULBP6-02 for 24 hours. Data represent mean ± SD of three technical replicates per condition from one of four biological replicates and were analyzed using one-way ANOVA for statistical significance. **D,** Cell surface NKG2D expression on human NK (CD45^+^ CD3^–^ CD56^+^) cells or CD8^+^ T (CD45^+^ CD3^+^ CD56^–^ CD8^+^) cells analyzed from IL-2/IL-15–primed PBMCs cultured with 50 nmol/L recombinant sULBP6-02 or PBS. Data represent mean ± SD of three technical replicates per condition from one of two biological replicates. Welch’s *t* test was used for statistical analysis. **E,** Quantification of COV644-GFP cell growth, as measured by GFP area per well by an Incucyte live cell analysis system, in the presence of IL-2/IL-15–primed PBMCs and 100 nmol/L recombinant sULBP6-02. Quantification is represented continuously over a 5-day time course. Data represent mean ± SD of three technical replicates per condition from one of four biological replicates. The last time point was analyzed using an unpaired *t* test for statistical significance. **F,** μMT^−/−^ mice were inoculated with syngeneic MC38 EV or MC38 ULBP6-02 cells by subcutaneous injection (*n* = 10 mice per group). Nineteen days after inoculation, tumors were resected and processed for flow cytometric analysis. Bar graphs show the percentage of total tumor-infiltrating CD45^+^, NK (CD45^+^ Thy1.2^–^ NK1.1^+^), NKT (CD45^+^ Thy1.2^+^ NK1.1^+^), and CD8^+^ T (CD45^+^ Thy1.2^+^ NK1.1^–^ CD8^+^) cells and the percentage of CD25^+^ NK and NKT cells. Data represent mean ± SEM from one of two independent experiments and were analyzed using Welch’s *t* test for statistical significance. *, *P* ≤ 0.05; **, *P* ≤ 0.01; ***, *P* ≤ 0.001; ****, *P* ≤ 0.0001. ns, not significant.

We evaluated the binding affinities of all human NKG2DLs to NKG2D to further understand the role of ULBP6 in regulating antitumor immunity. Among all human NKG2DLs, the two most prevalent ULBP6 allelic isoforms, ULBP6-01 and ULBP6-02, exhibited the highest binding affinities to NKG2D, consistent with a previous report (Fig. 3A; Supplementary Table S4; ref. 48). This suggests that ULBP6, and particularly ULBP6-02, may be the most potent activator of NKG2D in the membrane-anchored form or the most potent suppressor in the soluble form.

### Soluble ULBP6 exerts potent immunosuppression by impeding the interaction between membrane-anchored NKG2DLs and NKG2D

Subsequent functional characterization of sULBP6 was performed using sULBP6-02 as a proof of concept, given its highest affinity to NKG2D. To assess the immunosuppressive capacity of sULBP6, we cocultured NKG2D-expressing PBMCs (Supplementary Fig. S3A and S3B) with COV644, a human ovarian cancer cell line that endogenously expresses membranous MICA/B and ULBP6 and produces sULBP6 (Supplementary Fig. S3C–S3F) in the presence of recombinant sULBP6 or sMICA, the latter of which has the third highest binding affinity to NKG2D of all NKG2DLs (Fig. 3A). Both sNKG2DLs resulted in a concentration-dependent reduction in PBMC-mediated IFNγ secretion; however, sULBP6 showed significantly greater suppression of IFNγ secretion compared with sMICA (Fig. 3B; Supplementary Fig. S4A; Supplementary Table S5), which reflects its higher affinity for NKG2D.

We next sought to investigate if sULBP6 induces immunosuppression by outcompeting membrane-anchored NKG2DLs. To do so, we cultured PBMCs on MICA-coated plates to mimic membrane-anchored MICA, with or without sULBP6. MICA alone significantly induced PBMC-mediated IFNγ secretion compared with that of ULBP4, a previously reported low-affinity NKG2DL (49), which served as a negative control as it did not exhibit binding to NKG2D in our assay system (Fig. 3C). The addition of sULBP6 completely reversed MICA-induced PBMC activation, which could be attributed to the high binding affinity of ULBP6 relative to MICA that allows sULBP6 to outcompete MICA for binding to NKG2D (Fig. 3C; Supplementary Table S5). The immunosuppression induced by sULBP6 was also associated with reduced detection of cell surface NKG2D on NK and CD8^+^ T cells (Fig. 3D; Supplementary Table S5). This may be due to direct competition between sULBP6 and the anti-NKG2D flow cytometry antibody for NKG2D or the induction of NKG2D internalization by sULBP6, as previously reported (15). In either scenario, the amount of cell surface NKG2D available to bind activating NKG2DLs is reduced.

The immunosuppression of PBMCs mediated by sULBP6 resulted in enhanced tumor cell growth, implying that sULBP6 hinders the ability of immune cells to control tumor cell growth (Fig 3E; Supplementary Fig. S4B and S4C). Collectively, these findings underscore the immunosuppressive effect of sULBP6, which exerts its inhibitory function even in the presence of membrane-anchored NKG2DLs.

### ULBP6 overexpression creates an immunosuppressive tumor microenvironment

Given that membrane-anchored sNKG2DLs have opposing effects on NKG2D activation and that ULBP6 exists in both forms in patients with cancer, we sought to understand the net effect of membrane and sULBP6 on the tumor microenvironment *in vivo*. Due to the lack of ULBP6 conservation between humans and rodents (50), we engineered the MC38 murine colorectal cancer cell line to overexpress human ULBP6. Human and murine NKG2D were confirmed to bind human ULBP6 with comparable affinities (Supplementary Table S6). Cell surface expression of ULBP6 was confirmed on tumor cells after subcutaneous implantation into μMT^−/−^ mice (used to minimize the immunogenicity of human ULBP6), and sULBP6 in mouse sera was within the range of sULBPs found in human plasma from patients with cancer (Supplementary Fig. S5A and S5B). Overexpression of ULBP6 by MC38 tumor cells resulted in significantly decreased infiltration of CD45^+^ cells, including NK, NKT, and CD8^+^ T cells, into the tumor microenvironment and decreased expression of the activation marker CD25^+^ on tumor-infiltrating NK and NKT cells (Fig. 3F). Despite these changes in the immune cells, the overexpression of human ULBP6 did not affect tumor growth in the MC38 model, potentially attributable to the model’s nondependency on human ULBP6 for its growth (Supplementary Fig. S5C) or redundancy of ULBP6 with the expression of endogenous murine NKG2DLs such as Rae-1 (51). Nonetheless, these results suggest that sULBP6 and/or chronic NKG2D stimulation (54) by membrane-associated ULBP6 derived from ULBP6 overexpression may counteract the immune-activating effects of membrane-anchored NKG2DLs, resulting in NK cell dysfunction (52) and an immunosuppressive tumor microenvironment. The totality of these results, the prevalence of sULBP6 in human tumors, and the immunosuppressive capacity of sULBP6 suggest that preventing sULBP6 from binding NKG2D may promote NK cell–mediated immuno surveillance and be a potential therapeutic intervention for cancer.

### 23ME-01473, an Fc-enhanced anti-ULBP6/2/5 antibody, binds ULBP6, ULBP2, and ULBP5 to block their binding to NKG2D

To this end, we developed 23ME-01473, an Fc-enhanced antibody with an afucosylated Fc domain (53), that binds strongly to ULBP6 with picomolar affinity (Tables 1 and 2). Given the high amino acid sequence homology between ULBP6 and its other family members (Supplementary Fig. S6A), we tested the cross-reactivity of 23ME-01473 to ULBP1, ULBP2, ULBP3, and ULBP5. High-affinity binding was observed between 23ME-01473 and the two closest related family members, ULBP2 and ULBP5 (Table 1). Furthermore, 23ME-01473 blocked the binding of the highest-affinity ULBPs, ULBP6, and ULBP5 to NKG2D, as demonstrated by an ELISA-based blocking assay (Table 1; Supplementary Fig. S6B and S6C).

**Table 1:** Characterization of the binding and blocking activity of 23ME-01473

	Binding Affinity of 23ME-01473 to ULBPs	Blockade of ULBPs to NKG2D by 23ME-01473
Ligand	K_D_ (nmol/L) ± SD (*n* = 4)	IC_50_ (nmol/L) ± SD (*n* = 4)
ULBP1	N.B.	N.A.
ULBP2	0.23 ± 0.009	N.A.
ULBP3	N.B.	N.A.
ULBP4	N.B.	N.A.
ULBP5	1.92 ± 0.071	0.31 ± 0.037
ULBP6	0.053 ± 0.006	0.04 ± 0.024

Abbreviations: N.A., not available; N.B., no binding detected.

**Table 2:** Binding affinity of anti-ULBP6/2/5 antibodies to human Fcγ receptors

	Fc-WT-anti-ULBP6/2/5	23ME-01473
Ligand	K_D_ (nmol/L) ± SD (*n* = 4)	K_D_ (nmol/L) ± SD (*n* = 4)
FcγRI (activating)	1.01 ± 0.12	0.92 ± 0.11
FcγRIIa-H_167_ (activating)	558 ± 5.60	270 ± 19.20
FcγRIIa-R_167_ (activating)	971 ± 13.90	406 ± 29.70
FcγRIIb (inhibitory)	2420 ± 42.1	2230 ± 351
FcγRIIIa-V_176_ (activating)	537 ± 14.30	6.74 ± 0.19
FcγRIIIa-F_176_ (activating)	1780 ± 116	25.50 ± 15.60

**Figure 4:**
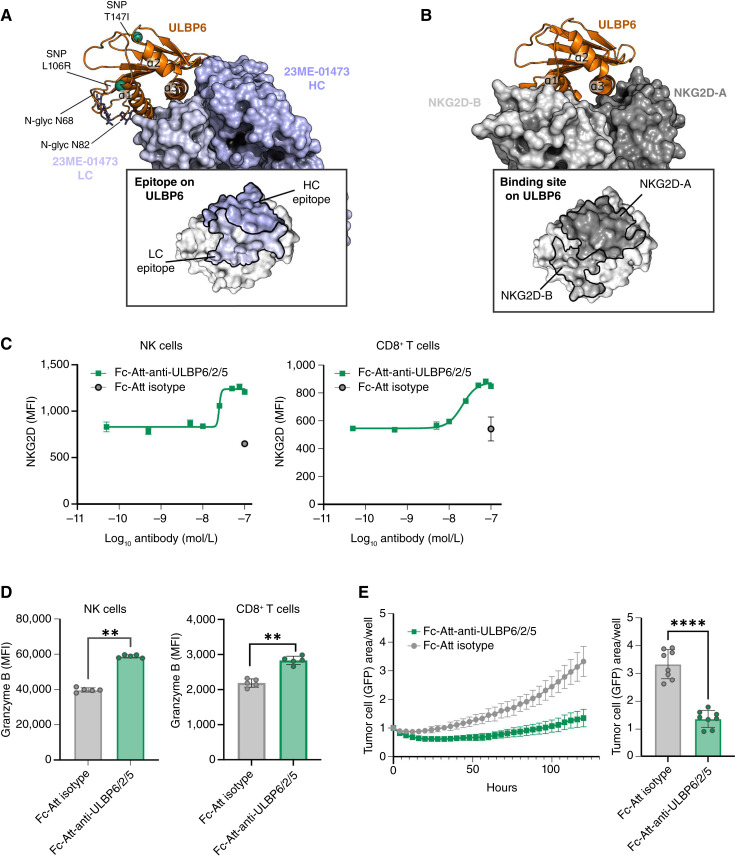
Neutralization of soluble ULBP6 by an anti-ULBP6/2/5 antibody promotes NK and CD8^+^ T cell activation and PBMC-mediated tumor killing. **A,** Crystal structure of 23ME-01473 Fab in complex with human ULBP6-02. The extracellular domain of ULBP6-02 is colored orange, glycan moieties from N-glycosylated asparagine are shown as sticks, and the coding single-nucleotide polymorphism regions are shown as green spheres. 23ME-01473 Fab is shown in surface representation with the light chain colored light purple and the heavy chain colored dark purple. **B,** Crystal structure of NKG2D in complex with human ULBP6-02 (PDB 4S0U). The two NKG2D protomers, NKG2D-A and NKG2D-B, of the dimer are shown in light and dark gray in surface representation, and ULBP6 is in the same orientation as in **A**, with its extracellular domain colored orange. **C,** Flow cytometric analysis of cell surface NKG2D expression on NK cells (CD45^+^ CD3^–^ CD56^+^) or CD8^+^ T cells (CD45^+^ CD3^+^ CD56^–^ CD8^+^) analyzed from IL-2/IL-15–primed PBMCs cultured with 50 nmol/L recombinant sULBP6-02 and 0.05–100 nmol/L Fc-Att-anti-ULBP6/2/5 or 100 nmol/L Fc-Att isotype control. Data represent mean ± SEM of three technical replicates per condition from one of four biological replicates. **D,** Flow cytometric analysis of granzyme B expression in NK cells (CD45^+^ CD3^–^ CD56^+^) or CD8^+^ T cells (CD45^+^ CD3^+^ CD56^–^ CD8^+^) analyzed from IL-2/IL-15–primed PBMCs cultured for 48 hours with COV644 cells, 50 nmol/L recombinant sULBP6-02, and 200 nmol/L Fc-Att-anti-ULBP6/2/5 or Fc-Att isotype control. Data represent mean ± SD of five technical replicates per condition from one of two biological replicates. The Mann–Whitney test was used for statistical analysis. **E,** Quantification of COV644-GFP cell growth, as measured by GFP area per well using an Incucyte live cell analysis system, in the presence of IL-2/IL-15–primed PBMCs, 50 nmol/L recombinant sULBP6-02, and 100 nmol/L Fc-Att-anti-ULBP6/2/5 or Fc-Att isotype control. Quantification is represented continuously over a 5-day time course (left) and at the end of the 5-day time point (right). Data represent mean ± SD of eight technical replicates per condition from one of four biological replicates. Welch’s *t* test was used for statistical analysis. **, *P* ≤ 0.01; ****, *P* ≤ 0.0001. HC, heavy chain; LC, light chain; MFI, mean fluorescence intensity; SNP, single-nucleotide polymorphism.

We then crystallized and solved the structure of the complex of the Fab domain of 23ME-01473, termed 23ME-01473 Fab, and ULBP6 at a resolution of 2.3Å to understand how 23ME-01473 blocks the interaction between ULBP6 and NKG2D (Fig. 4A; Supplementary Tables S7 and S8). Our complex is similar to a previously solved complex, with the exception of the glycosylation of residues N68 and N82 of ULBP6, likely due to the mammalian expression system used in this study (48). Most of the 23ME-01473 paratope is formed by ULBP6 residues located in helices α1 and α3, which form most of the NKG2D-binding site (Fig. 4A and B). Compared with the NKG2D-binding site, the 23ME-01473 Fab–binding site is shifted and thus not affected by ULBP6 polymorphisms at positions L106R (rs1555696) and T147I (rs61730071). Nevertheless, the structural paratope of 23ME-01473 and the NKG2D-binding site on ULBP6 significantly overlap. NKG2D is a dimeric protein composed of two protomers, A and B. The 23ME-01473 paratope on ULBP6 overlaps with most of the binding site of the NKG2D A protomer, demonstrating that 23ME-01473 blocks the ULBP6 interaction with NKG2D by direct competition. The structure of 23ME-01473 Fab in complex with ULBP6 also gives insight into the cross-reactivity of 23ME-01473 to ULBP2 and ULBP5. Although the 23ME-01473 paratope is highly conserved among ULBP6, ULBP2, and ULBP5 (94%), the conservation is much lower among ULBP6 and ULBP1, ULBP3, and ULBP4 (45%, 36%, and 24%, respectively; Supplementary Fig. S6D). Taken together, these data show that 23ME-01473 is a high-affinity antibody that blocks the binding of ULBP6, ULBP2, and ULBP5 to NKG2D.

### Blocking of soluble ULBP6 with Fc-Att-anti-ULBP6/2/5 reverses immune suppression

To assess the ability of 23ME-01473 to restore NKG2D activation by blocking sULBP6-mediated immunosuppression, we generated an effector-attenuated version of the antibody, termed Fc-Att-anti-ULBP6/2/5, which does not activate Fc receptors. Fc-Att-anti-ULBP6/2/5 reversed sULBP6-mediated reduction of NKG2D on NK and CD8^+^ T cells in a concentration-dependent manner (Fig. 4C; Supplementary Table S9). Additionally, Fc-Att-anti-ULBP6/2/5 rescued IFNγ production from PBMCs cocultured with COV644 cells (Supplementary Fig. S7A; Supplementary Table S9) and significantly augmented granzyme B and Ki-67 expression in NK and CD8^+^ T cells (Fig. 4D; Supplementary Fig. S7B; Supplementary Table S9), which was recapitulated with PBMCs cultured with NKG2D-activating plate-bound MICA and Fc-Att-anti-ULBP6/2/5 (Supplementary Fig. S7C–S7E). Blocking the immunosuppressive effects of sULBP6 also resulted in enhanced PBMC-mediated tumor cell killing (Fig. 4E; Supplementary Table S9), suggesting that Fc-Att-anti-ULBP6/2/5 can restore PBMC-mediated tumor growth control. Furthermore, this effect was replicated with NK cells, suggesting that the antitumor effect of sULBP6 neutralization via Fc-Att-anti-ULBP6/2/5 may be driven at least partially through NK cells (Supplementary Fig. S7F).

### Dual NKG2D and FcγRIIIa activation enhances immune cell activation

**Figure 5:**
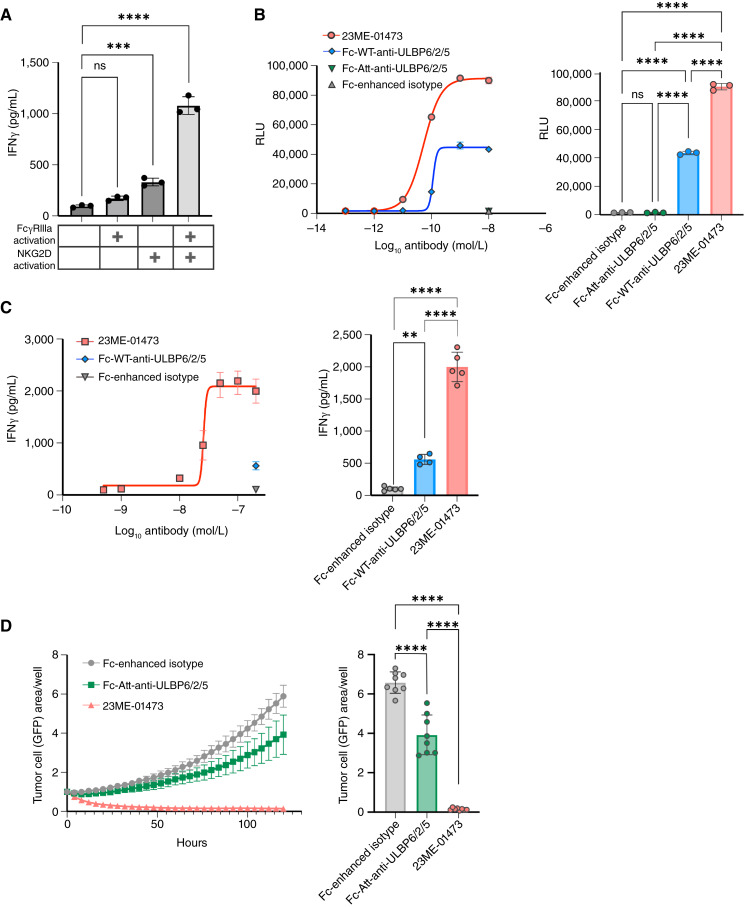
Dual FcγRIIIa and NKG2D activation by 23ME-01473, an Fc effector–enhanced anti-ULBP6/2/5 antibody, induces augmented antitumor immunity. **A,** IFNγ concentration of the supernatants of IL-2/IL-15–primed PBMCs cultured with plate-bound antibodies that activate FcγRIIIa (Fc-WT-anti-lysozyme antibody; X-lys-E+), NKG2D (Fc-Att-anti-lysozyme antibody fused to ULBP6-02; ULBP6-lys-E−), both receptors (Fc-WT-anti-lysozyme antibody with fused ULBP6-02; ULBP6-lys-E+), or neither receptor (Fc-Att-anti-lysozyme antibody; X-lys-E−) for 24 hours. Data represent mean ± SD of three technical replicates per condition from one of four biological replicates. **B,** FcγRIIIa activation as measured by RLU using a Promega ADCC assay with a luciferase effector cell line, COV644 cells, and 0.0001 to 10 nmol/L Fc-WT-anti-ULBP6/2/5 or 23ME-01473, or 10 nmol/L Fc-Att-anti-ULBP6/2/5 or Fc-enhanced isotype control, is depicted as a concentration-dependent response (left) or in response to 10 nmol/L of the indicated antibodies (right). Data represent mean ± SD of three technical replicates per condition from one of four biological replicates. **C,** IFNγ concentration of the supernatants of IL–2/IL-15–primed PBMCs cultured with COV644 cells, 50 nmol/L recombinant sULBP6-02, and 0.05–200 nmol/L 23ME-01473, or 200 nmol/L Fc-WT-anti-ULBP6/2/5 or Fc-enhanced isotype control, is depicted as a concentration-dependent response (left) or in response to 200 nmol/L of the indicated antibodies (right). Data represent mean ± SD of five technical replicates per condition from one of four biological replicates. **D,** Quantification of COV644-GFP cell growth, as measured by GFP area per well by an Incucyte live cell analysis system, in the presence of IL-2/IL-15–primed PBMCs, 50 nmol/L recombinant sULBP6-02, and 100 nmol/L 23ME-01473, Fc-Att-anti-ULBP6/2/5, or Fc-enhanced isotype control. Quantification is represented continuously over a 5-day time course (left) and at the end of the 5-day time point (right). Data represent mean ± SD of eight technical replicates per condition from one of four biological replicates. One-way ANOVA was used for statistical analyses. **, *P* ≤ 0.01; ****, *P* ≤ 0.0001. ns, not significant; RLU, relative light unit.

In addition to the soluble form, ULBP6 can also be found in the membrane-anchored form on cancer cells. Given this and the near-exclusive expression of ULBP6/2/5 on malignant cells in the tumor microenvironment, we hypothesized that leveraging the Fc domain of an anti-ULBP6/2/5 antibody to augment ADCC upon binding to ULBP6/2/5-expressing cancer cells may further enhance antitumor immunity. Moreover, previous reports suggest that NKG2D may serve as a master regulator by amplifying the signaling of key NK cell–activating receptors, such as FcγRIIIa, by reducing their activation thresholds (54, 55). To investigate the cross-talk between NKG2D and FcγRIIIa, we created four tool reagents that activate FcγRIIIa, NKG2D, both FcγRIIIa and NKG2D, or neither FcγRIIIa nor NKG2D (Supplementary Fig. S8). As expected, activation of FcγRIIIa or NKG2D moderately increased PBMC activation, as assessed by IFNγ production (Fig. 5A). However, activation of both FcγRIIIa and NKG2D simultaneously led to a significant increase in IFNγ production, which was greater than that induced by each activating receptor individually. These data suggest that although activation through either FcγRIIIa or NKG2D can drive a strong immune response, stimulating both simultaneously can promote a stronger immune activation.

### NKG2D and FcγRIIIa activation by 23ME-01473 reverses immune suppression *in vitro*

Given that concurrent activation of FcγRIIIa and NKG2D enhances immune cell activation (Fig. 5A), we hypothesized that 23ME-01473, an anti-ULBP6/2/5 with an effector-enhanced (afucosylated) Fc domain (53), could capitalize on the presence of ULBP6/2/5 on the surface of tumor cells to activate FcγRIIIa and induce ADCC while concurrently neutralizing sULBP6 to release NKG2D to bind activating surface NKG2DLs. We evaluated the FcγRIIIa engagement capacity of three anti-ULBP6/2/5 variants: Fc-Att-anti-ULBP6/2/5 (effectorless, no FcγRIIIa stimulation), Fc-WT-anti-ULBP6/2/5 (WT Fc, moderate FcγRIIIa stimulation), and 23ME-01473 (effector-enhanced, strong FcγRIIIa stimulation). As expected, the effectorless Fc-Att-anti-ULBP6/2/5 exhibited no FcγRIIIa activation, whereas Fc-WT-anti-ULBP6/2/5 displayed significantly higher activation in a concentration-dependent manner. Compared with Fc-WT-anti-ULBP6/2/5, 23ME-01473 exhibited significantly higher induction of FcγRIIIa activation (Table 2), with an approximately twofold lower EC_50_ and twofold higher E_max_ (Fig. 5B; Supplementary Table S10).

We next explored whether the enhanced FcγRIIIa activation facilitated by 23ME-01473 translated to enhanced IFNγ secretion by NK cells by coculturing anti-ULBP6/2/5 antibodies with PBMCs and COV644s in the presence of sULBP6. Although Fc-WT-anti-ULBP6/2/5 induced greater IFNγ production than Fc-Att-anti-ULBP6/2/5 (Supplementary Fig. S9A), 23ME-01473 induced an even greater increase in IFNγ production compared with Fc-WT-anti-ULBP6/2/5 (Fig. 5C; Supplementary Table S10). This increase in immune cell activation mediated by 23ME-01473 translated to superior and, in some PBMC donors, rapid and nearly complete tumor cell killing (Fig. 5D; Supplementary Fig. S9B; Supplementary Table S10). Thus, NKG2D activation through sULBP6 blockade and FcγRIIIa engagement, via enhanced binding affinity for FcγRIIIa and/or bridging of FcγRIIIa to ULBP6/2/5, each contribute to antitumor immunity, but simultaneous activation with an Fc-effector-enhanced anti-ULBP6/2/5 amplifies this effect.

### 23ME-01473 exerts tumor growth control *in vivo*

**Figure 6:**
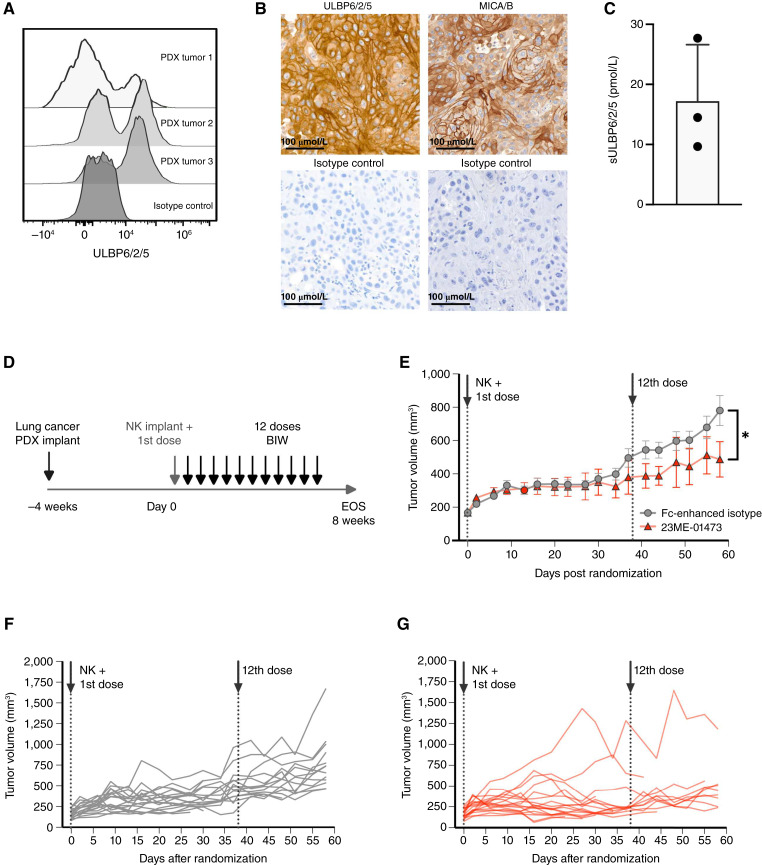
23ME-01473 significantly inhibits the growth of a NSCLC patient-derived xenograft cancer model *in vivo.***A,** Cell surface expression of ULBP6/2/5 on EpCAM^+^ tumor cells from the CTG-3470 NSCLC PDX cell line implanted and grown in NOG mice as measured by flow cytometry. Data from three biological replicates are shown. **B,** Representative IHC images of ULBP6/2/5 or MICA/B expression (top) and the corresponding isotype controls (bottom) in CTG-3470 NSCLC PDX resected tumors. **C,** Soluble ULBP6/2/5 detected from the sera of three biological replicates. Data represent mean ± SD. **D,** Experimental design of *in vivo* efficacy study. **E,** Growth curves of NSCLC PDX tumors implanted in NOG mice treated with 10 mg/kg BIW 23ME-01473 or isotype control. Data represent mean ± SEM of 18 mice per group. Statistical analysis was performed using a one-sided test to determine if the ratio of mean adjusted area under the curve between 23ME-01473 and control groups was significantly less than 1, indicating lower tumor growth, with significance assessed at an FDR-adjusted alpha level of 0.05. Individual tumor spider plots for (**F**) isotype control or (**G**) 23ME-01473. Dotted lines indicate the day of NK cell infusion and the first and last doses of 23ME-01473 or isotype control. *, *P* ≤ 0.05. BIW, twice a week; EOS, end of study.

The ability of 23ME-01473 to activate NK cells and induce tumor growth control *in vivo* was evaluated using a humanized NSCLC PDX model that endogenously expresses cell surface ULBP6/2/5 and MICA/B (Fig. 6A and B) and produces sULBP6/2/5 (Fig. 6C). The endogenous expression of human NKG2DLs in this PDX model and FcγRIIIa on engrafted human NK cells allows for the evaluation of the antitumor activity of 23ME-01473 in a more physiologically relevant system. In mice implanted with NSCLC and human NK cells (Fig. 6D), 23ME-01473 administration resulted in a significant 48% tumor growth inhibition compared with the isotype control (Fig. 6E). Furthermore, tumors from individual mice in the control arm exhibited faster growth kinetics (Fig. 6F) compared with those in the 23ME-01473 arm (Fig. 6G), which is most apparent near the end of the dosing regimen. This delayed difference in tumor growth inhibition between the two treatment arms could be attributed to the inherently slow growth of the PDX model, delayed accumulation of soluble and/or membrane-associated ULBP6/2/5, and/or delayed NK cell proliferation in the 23ME-01473 treatment arm. Although the relative contribution of NKG2D or FcγRIIIa activation to antitumor activity is difficult to resolve in this model, simultaneous activation of both receptors can reinvigorate NK cell–mediated antitumor immunity.

## Discussion

ICIs, such as anti–PD-L1 and anti–CTLA-4, have revolutionized cancer treatment, yet many patients do not derive benefit from these therapies, and novel immunotherapies often fail in clinical development. Incorporating human genetics early into drug discovery and development can improve the likelihood of clinical success for novel drugs (28, 29). By leveraging 23andMe, Inc.’s large-scale germline genetic database, we identified *RAET1L* (ULBP6) as a promising I/O drug target, given its inverse risk associations with cancer and immunologic disorders.

Although agents targeting the NKG2D/NKG2DL axis are currently in clinical development, none specifically target ULBP6 (55). The near-exclusive expression of *RAET1L* (ULBP6) in malignant cells within the tumor microenvironment distinguishes it from nontumor-specific NKG2DLs such as MICA/B, which may translate to an improved therapeutic window for ULBP6-targeting therapies. Moreover, ULBP6 exhibits the highest-binding affinity to NKG2D among all NKG2DLs, which suggests that it may be the most potent activating ligand for NKG2D when in the membrane-associated form and, conversely, the most potent suppressive ligand in the soluble form. Indeed, our work indicates that sULBP6 can outcompete the binding of membrane-anchored activating NKG2DLs, thereby suppressing immune activation and tumor cell killing, consistent with the function of other human sNKG2DLs (13–15). Thus, ULBP6 may be the cornerstone of NKG2D axis-targeting therapies. Given this, we developed 23ME-01473, an anti-ULBP6/2/5 antibody that relieves NKG2D suppression by blocking sULBP6/2/5 and engages membrane-associated ULBP6/2/5 and FcγRIIIa to induce ADCC to augment antitumor immunity. Importantly, our data suggest that targeting tumors harboring at least one allele of the high-affinity ULBP6-02 isoform, which may be the most immunosuppressive isoform in the soluble form, may elicit the greatest antitumor activity with 23ME-01473. However, the clinical activity of 23ME-01473 may not be limited to individuals carrying the ULBP6-02 isoform. Therefore, monitoring patients’ ULBP6 haplotype, in addition to tumor and peripheral ULBP6 levels, is warranted during the clinical evaluation of 23ME-01473 and may be important for other therapeutics targeting this pathway.

Although we use controlled and reductionist systems to demonstrate the activity and potency of sULBP6 and 23ME-01473, further work is required to validate these findings in more complex models. For example, although we show the immunosuppressive effects of sULBP6 using recombinant monomeric sULBP6 *in vitro*, sNKG2DLs can be generated through two mechanisms—proteolytic cleavage from the cell surface and exosomal release (Supplementary Fig. S10; refs. 56–58)—which may have differential potency for NKG2D-binding. Exosomes can cluster multiple ligand molecules on their cell surface, which would result in increased receptor avidity of exosomal ligands compared with monomeric ligands (59, 60). Accordingly, exosomal ULBP6 may impart a different function on NKG2D than monomeric sULBP6. The contribution of either sULBP6 species in terms of their concentration and immunosuppressive potency in the periphery and especially the tumor microenvironment is currently unknown and warrants further investigation to fully understand the mechanism of sULBP6-mediated immunosuppression. Additionally, although we show immunosuppressive effects of sULBP6, which are consistent with the role of sMICA/B (13, 14) and sULBP1-3 (15), we recognize that not all sNKG2DLs may function in the same way, especially in the *in vivo* setting. For example, sMULT1, a murine sNKG2DL that does not have a human equivalent, can reactivate NKG2D on NK cells by reversing the desensitization of NKG2D provided by membrane-associated RAE-1 expressed on tumor-associated cells (61, 62) *in vivo*. Our mRNA analysis of the tumor-associated cells in a human HNSCC cohort suggests that endothelial and fibroblast cells express ULBP6/2/5 at minimal to low levels, which may not be sufficient to chronically activate NKG2D. However, we cannot rule out that this mechanism observed in mice does not occur in other human tumors, which will require future investigation.

In evaluating the net effect of ULBP6 overexpression *in vivo*, we observed reduced infiltration of activated immune cells in MC38 syngeneic murine tumors that overexpress human ULBP6. This could be attributed to an accumulation of sULBP6 that suppresses NKG2D activation and/or membrane-associated ULBP6 that desensitizes NKG2D activation through chronic activation. Notably, the ectopic expression of human ULBP6 did not affect MC38 tumor growth, suggesting it is not necessary for MC38-derived tumor growth or may be redundant with endogenous murine NKG2DLs. This may be model-specific, as others have reported modulation of tumor growth and tumor-free survival by ectopic expression or overexpression of NKG2DLs in murine models of lymphoma and prostate cancer (63, 64). Nonetheless, the lack of conservation of human ULBP6 and MICA/B in mice and the species differences in FcγR expression and affinities to hIgG1 limit our ability to evaluate the role of ULBP6 and 23ME-01473-mediated NKG2D and FcγRIIIa activation on tumor growth in the MC38 model. To address this limitation, we employed a human NSCLC PDX model and demonstrated that 23ME-01473 enhances NK cell–mediated tumor growth control despite the absence of a fully intact immune system. Infusing only human NK cells enabled the evaluation of the direct effects of sULBP6 blockade and FcγRIIIa engagement on NK cell–mediated antitumor immunity but did not allow for the investigation of the possible effects driven by NKG2D-expressing T cells, which, together with NK cells, may have augmented the antitumor activity of 23ME-01473. Further investigations into the effect of NKG2D activation on T cells are warranted to understand the contribution of T cell–mediated antitumor immunity. Importantly, the modest 23ME-01473-mediated antitumor activity observed *in vivo* may not reflect its activity in human tumors, especially given the limitations of each model employed and the compelling genetic association of ULBP6 with cancer derived from our large-scale human genetic and health data.

Targeting NK cells through the NKG2D axis holds significant therapeutic potential, especially in cases of ICI resistance mediated by MHC class I downregulation or loss (1, 2), as NK cells can elicit MHC I–independent cytotoxicity. Furthermore, studies have demonstrated an increased presence of NK cells in *B2M*-LOH or *B2M*-heterozygous tumors compared with *B2M-*unaltered tumors (65, 66), suggesting that MHC class I deficiency may render tumors vulnerable to NK cell–mediated cytotoxicity. Recent reports also reveal that CD8^+^ T cells can be activated by MHC I–negative tumor cells through NKG2D engagement after prior antigen-specific activation by MHC I–positive tumor cells or adjacent myeloid antigen-presenting cells (5). Thus, reinvigorating the NKG2D axis may enhance both innate and adaptive antitumor immunity, even in the absence of MHC I. In our study, we demonstrate sULBP6-mediated immunosuppression of primary human NK and CD8^+^ T cells, and the ability of 23ME-01473 to block the sULBP6-NKG2D interaction and restore immune activation. These findings, combined with our current understanding of antitumor immunity in MHC I–negative tumors, suggest that 23ME-01473 may be an effective therapy in such tumor settings.

NKG2D plays an important role in NK cell responses, as it can augment the signaling of other NK cell receptors, including FcγRIIIa (67). Previous work has demonstrated the therapeutic benefit of engaging multiple NK cell–activating receptors, such as NKG2D and FcγRIIIa. For example, an antibody that blocks the shedding of MICA/B from tumor cells and induces ADCC and antibody-dependent cellular phagocytosis has shown early signs of clinical activity in patients with advanced solid tumors (68). It is hypothesized that the stabilization of MICA/B at the tumor cell surface not only prevents immune evasion through MICA/B shedding but also increases the target antigen density to enhance ADCC and antibody-dependent cellular phagocytosis. With 23ME-01473, we demonstrate that enhanced activation of FcγRIIIa amplifies NKG2D-mediated NK cell activation and tumor growth control. The high affinity of 23ME-01473 for ULBP6 suggests that it may predominantly target ULBP6, especially in the context of equivalent expression of ULBP6/2/5. However, the cross-reactivity of 23ME-01473 to ULBP6/2/5 presents an advantage in targeting an increased repertoire of membrane-associated NKG2DLs for improved ADCC induction and sNKG2DLs for enhanced NKG2D de-repression.

In totality, our results highlight the role of sULBP6 in mediating immunosuppression and the ability of 23ME-01473 to reinvigorate the NKG2D axis by blocking this immunosuppression and inducing ADCC by targeting cell surface ULBP6/2/5. The combined activation of NKG2D and FcγRIIIa receptors on NK cells underscores the distinct and potentially complementary mechanisms of 23ME-01473 as compared to those of ICIs currently used as standard of care. 23ME-01473 is currently being evaluated in patients with advanced malignancies in a first-in-human, phase I clinical trial (NCT06290388), in which target saturation, dosing levels and frequency, and efficacy will be determined.

## Supplementary Material

Supplementary Figure S1 Supplementary Figure S1

Supplementary Figure S2 Supplementary Figure S2

Supplementary Figure S3 Supplementary Figure S3

Supplementary Figure S4 Supplementary Figure S4

Supplementary Figure S5 Supplementary Figure S5

Supplementary Figure S6 Supplementary Figure S6

Supplementary Figure S7 Supplementary Figure S7

Supplementary Figure S8 Supplementary Figure S8

Supplementary Figure S9 Supplementary Figure S9

Supplementary Figure S10 Supplementary Figure S10

Supplementary Tables S1-S10 Supplementary Tables S1-S10

Supplementary Data List of collaborators from 23andMe Research Team
