# Supplementary material for: 23ME-01473, an Fc Effector–Enhanced Anti-ULBP6/2/5 Antibody, Restores NK Cell–Mediated Antitumor Immunity through NKG2D and FcγRIIIa Activation
Source: Cancer Res Commun. 2025 Mar 21;5(3):477–96. doi: 10.1158/2767-9764.CRC-24-0478 (PMC11927390; doi:10.1158/2767-9764.CRC-24-0478)
Supplement: Supplementary Figure S1 [file crc-24-0478_supplementary_figure_s1_suppsf1.pdf]

Supplementary Figure S1

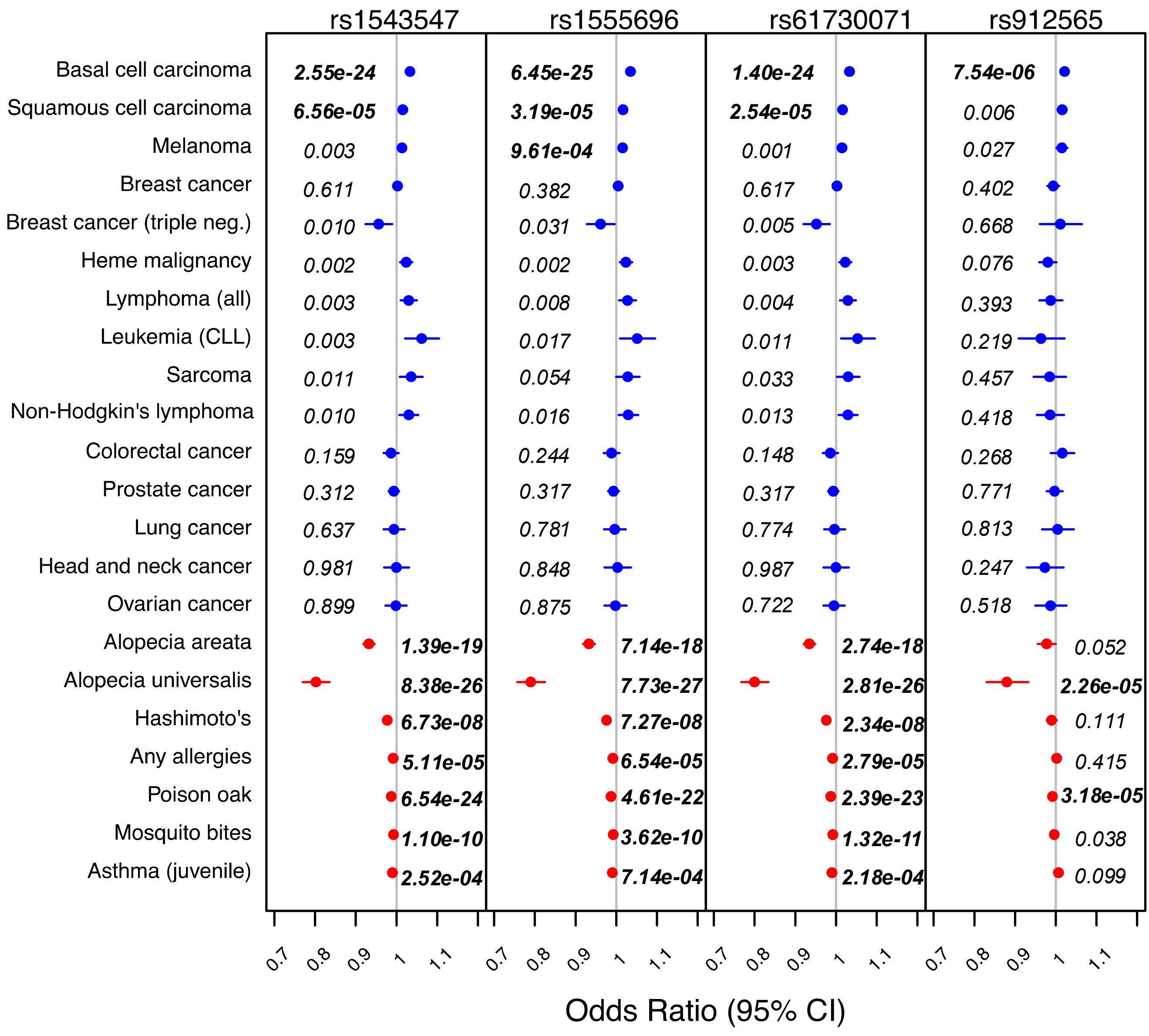

Supplementary Figure S1: The association of the four *RAET1L* variants with additional cancer phenotypes

The association of the four *RAET1L* variants shown in the credible set, as shown in Figure 1C, with additional cancers (shown in blue) that exhibit similar directionality as basal cell carcinoma. P values are annotated in italics; statistically significant P values are annotated in bold text.
