# Supplementary material for: 23ME-01473, an Fc Effector–Enhanced Anti-ULBP6/2/5 Antibody, Restores NK Cell–Mediated Antitumor Immunity through NKG2D and FcγRIIIa Activation
Source: Cancer Res Commun. 2025 Mar 21;5(3):477–96. doi: 10.1158/2767-9764.CRC-24-0478 (PMC11927390; doi:10.1158/2767-9764.CRC-24-0478)
Supplement: Supplementary Figure S2 [file crc-24-0478_supplementary_figure_s2_suppsf2.pdf]

Supplementary Figure S2

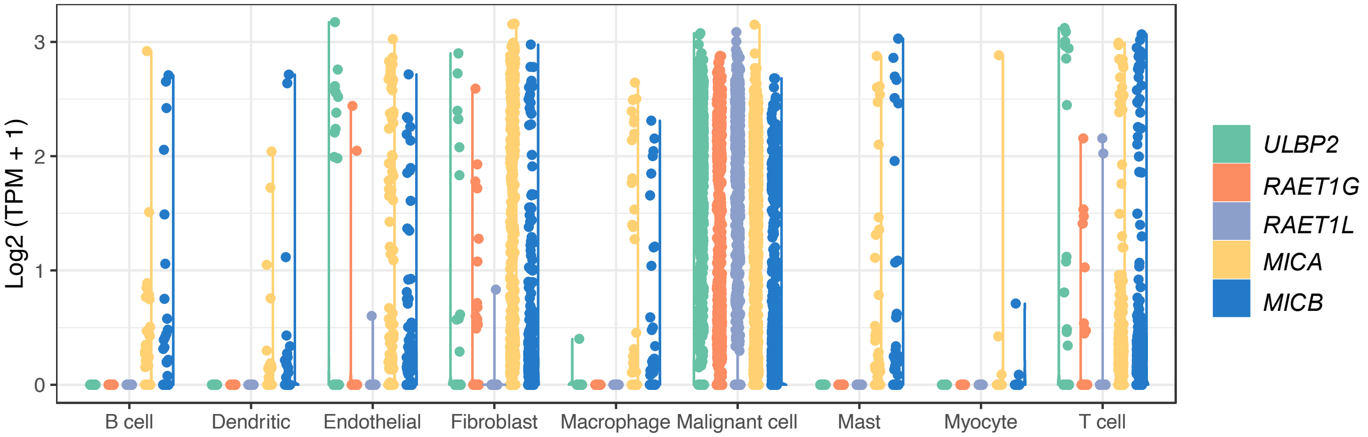

Supplementary Figure S2: *ULBP2*, *RAET1G*, *RAET1L*, and *MICA/B* mRNA expression in HNSC tumors

mRNA expression of *ULBP2*, *RAET1G*, *RAET1L, MICA*, and *MICB* in stromal, immune, and malignant cancer cells (N=5,902) from 18 treatment-naive HNSC patients.
