# Supplementary material for: 23ME-01473, an Fc Effector–Enhanced Anti-ULBP6/2/5 Antibody, Restores NK Cell–Mediated Antitumor Immunity through NKG2D and FcγRIIIa Activation
Source: Cancer Res Commun. 2025 Mar 21;5(3):477–96. doi: 10.1158/2767-9764.CRC-24-0478 (PMC11927390; doi:10.1158/2767-9764.CRC-24-0478)
Supplement: Supplementary Figure S3 [file crc-24-0478_supplementary_figure_s3_suppsf3.pdf]

Supplementary Figure S3

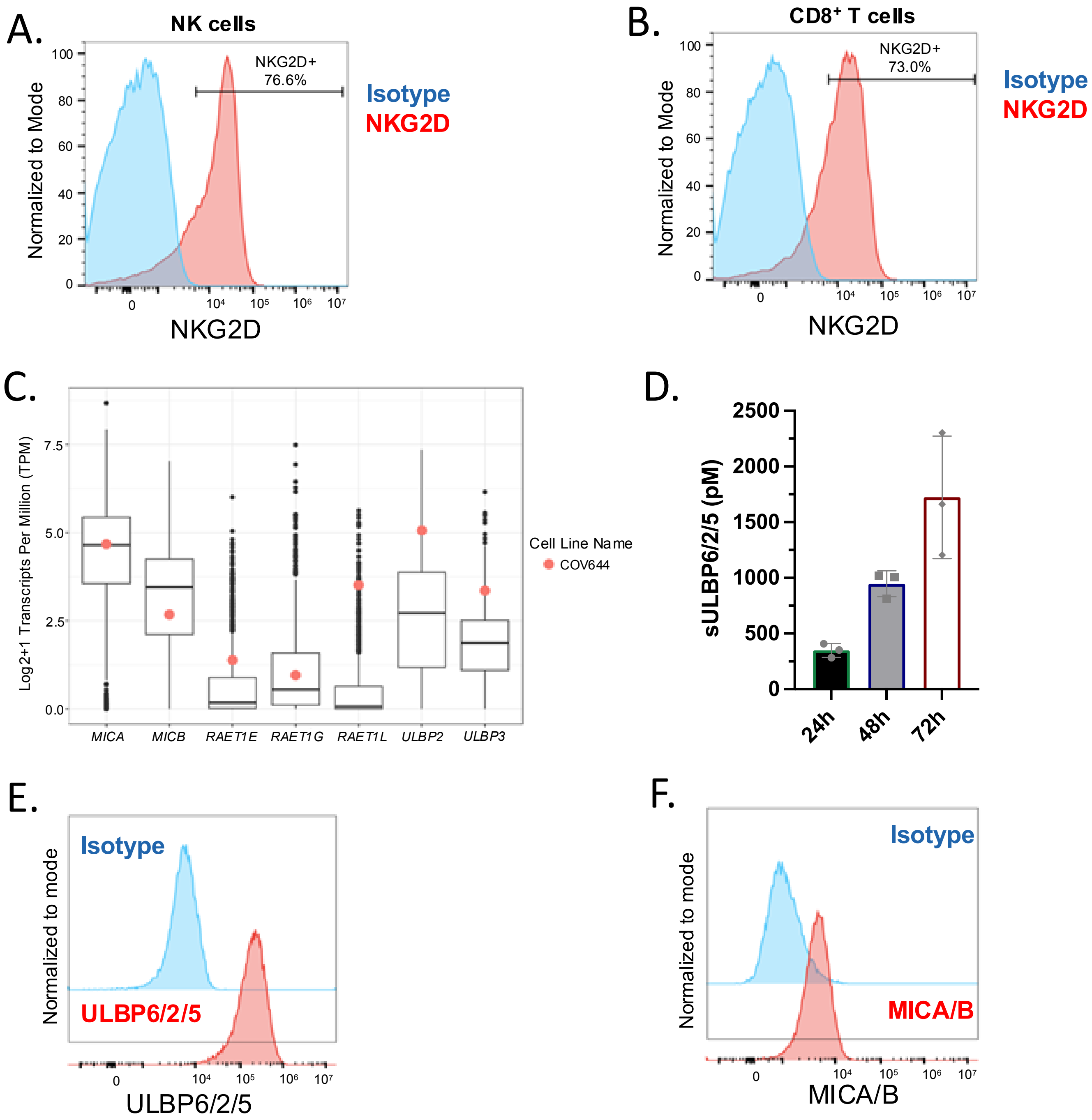

Supplementary Figure S3: Expression of NKG2DLs on COV644 cells

Human PBMCs were primed with IL-2/IL-15 for 36 hours and analyzed for NKG2D expression by flow cytometry on **A)** NK cells and **B)** CD8$^+$ T cells. Percent of positive expression is annotated in black text and lines in the histograms. Representative data from one biological replicate. **C)** mRNA expression of NKG2DLs in cancer cell lines from the Cancer Cell Line Encyclopedia (N=1,408), and specifically in the COV644 cell line annotated in the red-colored points. **D)** sULBP6/2/5 concentration of the supernatant of COV644 cells cultured for 24, 48, and 72 hours. Data represent mean ± SD for three biological replicates per condition. Cell surface expression of **E)** ULBP6/2/5 and **F)** MICA/B on COV644 cells as measured by flow cytometry and depicted as histograms. Abbreviation: TPM=transcripts per million.
