# Supplementary material for: 23ME-01473, an Fc Effector–Enhanced Anti-ULBP6/2/5 Antibody, Restores NK Cell–Mediated Antitumor Immunity through NKG2D and FcγRIIIa Activation
Source: Cancer Res Commun. 2025 Mar 21;5(3):477–96. doi: 10.1158/2767-9764.CRC-24-0478 (PMC11927390; doi:10.1158/2767-9764.CRC-24-0478)
Supplement: Supplementary Figure S4 [file crc-24-0478_supplementary_figure_s4_suppsf4.pdf]

Supplementary Figure S4

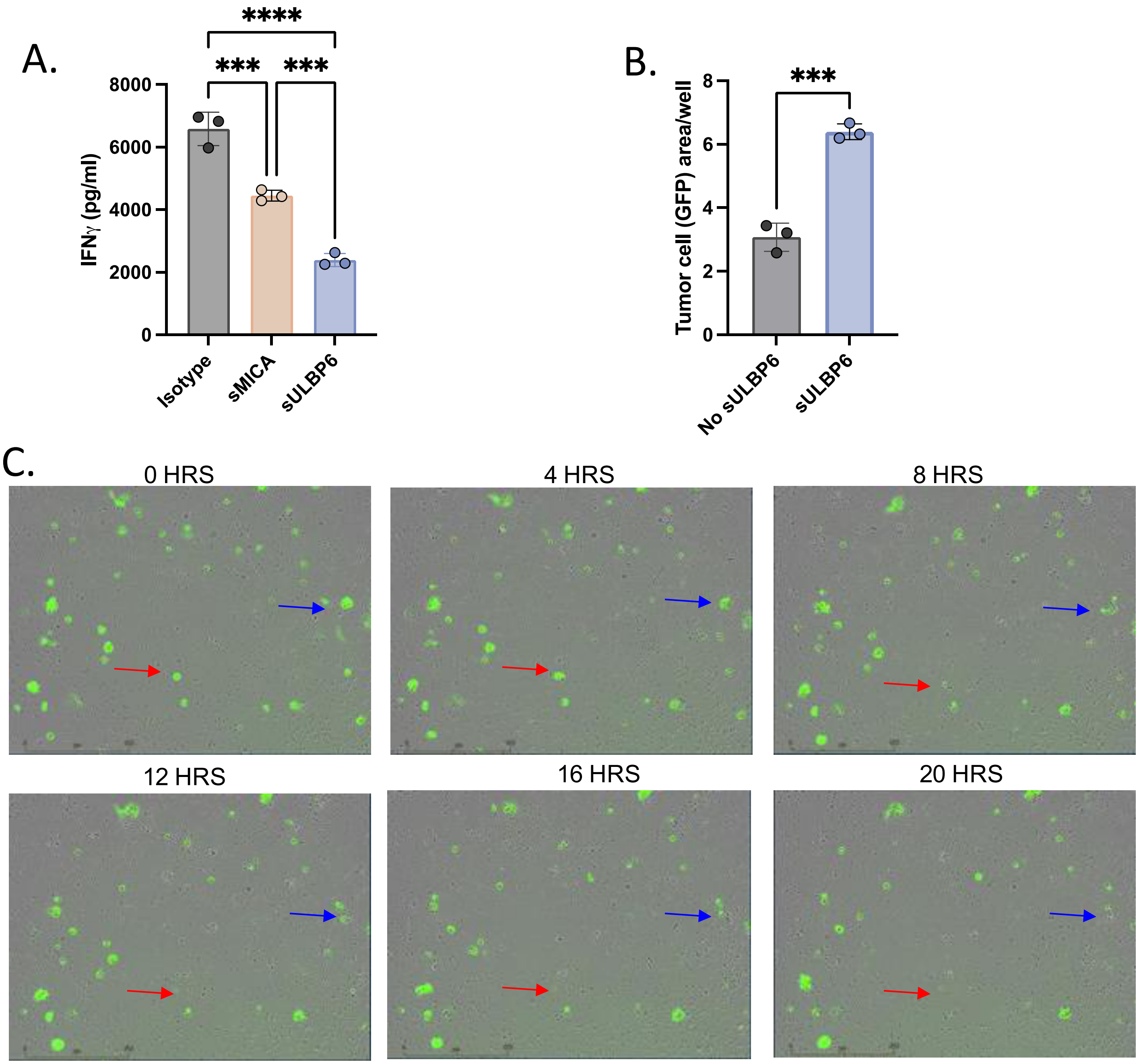

Supplementary Figure S4: sULBP6 reduces immune cell killing of GFP$^+$ COV644 cells
A) IFNγ concentration of the supernatants of IL-2/IL-15-primed PBMCs co-cultured with COV644 cells and 200 nM recombinant sULBP6-02, sMICA, or no ligand for 24 hours. Data represent mean ± SD of three technical replicates per condition from one of three biological replicates and were analyzed using one-way ANOVA for statistical significance. **B)** Quantification of COV644-GFP cell growth, as measured by GFP area per well in the presence of IL-2/IL-15-primed PBMCs and 100 nM recombinant sULBP6-02. Quantification is represented at the end of the 5 day timepoint. Data represent mean ± SD of three technical replicates per condition from one of four biological replicates, and unpaired t test was used for statistical significance. **C)** Representative images of PBMC-mediated tumor cell killing during 0 to 20 hours of 5 day time-course described in **B)**, with colored arrows pointing to the killing of two representative COV644-GFP cells. Data represent one of four biological replicates. ** $P \leq 0.01$, *** $P \leq 0.001$, **** $P \leq 0.0001$.
