# Supplementary material for: 23ME-01473, an Fc Effector–Enhanced Anti-ULBP6/2/5 Antibody, Restores NK Cell–Mediated Antitumor Immunity through NKG2D and FcγRIIIa Activation
Source: Cancer Res Commun. 2025 Mar 21;5(3):477–96. doi: 10.1158/2767-9764.CRC-24-0478 (PMC11927390; doi:10.1158/2767-9764.CRC-24-0478)
Supplement: Supplementary Figure S5 [file crc-24-0478_supplementary_figure_s5_suppsf5.pdf]

Supplementary Figure S5

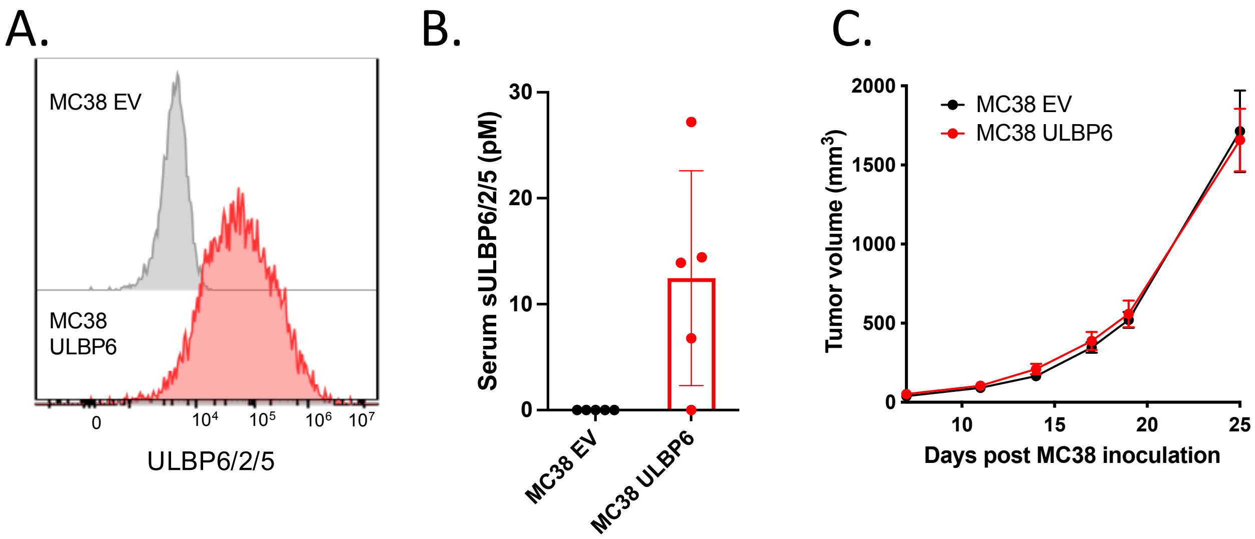

Supplementary Figure S5: MC38 syngeneic tumor model engineered to overexpress human ULBP6

The MC38 syngeneic murine tumor model was engineered to express either empty vector control (MC38 EV) or ULBP6 (MC38 ULBP6) using a lentiviral system. These tumor cell lines were implanted into μMt$^{-/-}$ mice and the overexpression of **A)** cell surface ULBP6/2/5 was measured by flow cytometry and **B)** serum sULBP6/2/5 by ELISA 19 days after inoculation . Data represent mean ± SEM for five mice per condition. **C)** Tumor growth curves of each model were measured. Data represent mean ± SEM from 17 mice per condition.
