# Supplementary material for: 23ME-01473, an Fc Effector–Enhanced Anti-ULBP6/2/5 Antibody, Restores NK Cell–Mediated Antitumor Immunity through NKG2D and FcγRIIIa Activation
Source: Cancer Res Commun. 2025 Mar 21;5(3):477–96. doi: 10.1158/2767-9764.CRC-24-0478 (PMC11927390; doi:10.1158/2767-9764.CRC-24-0478)
Supplement: Supplementary Figure S6 [file crc-24-0478_supplementary_figure_s6_suppsf6.pdf]

Supplementary Figure S6

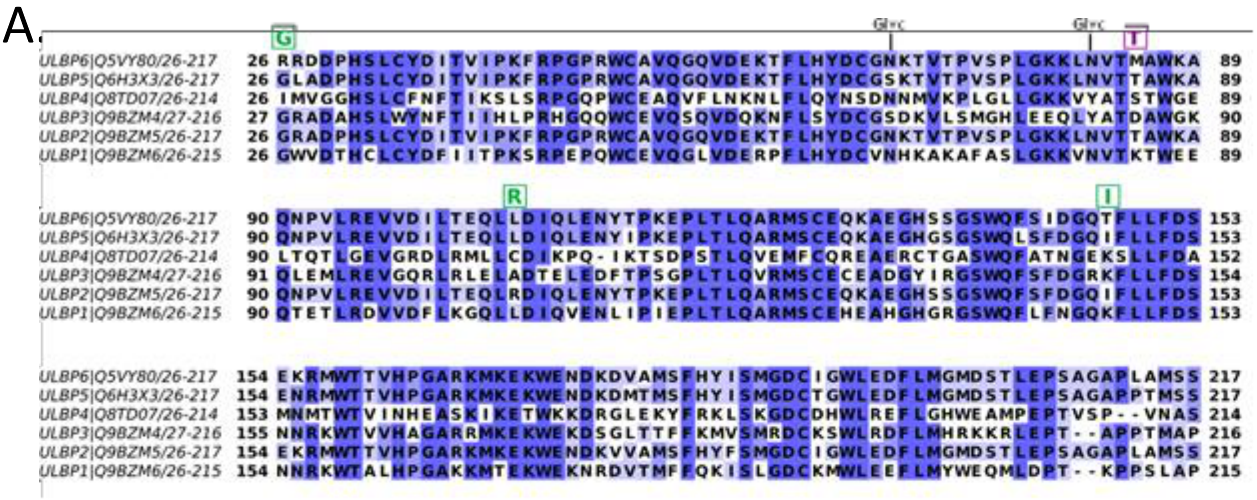

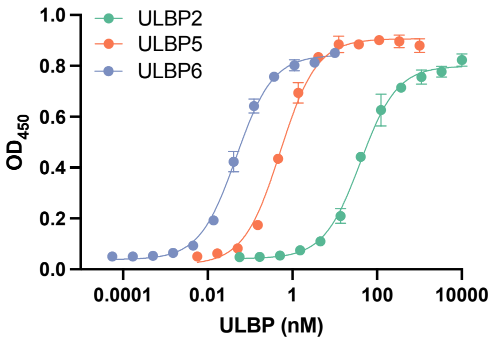

NKG2DL	EC80 (nM)
ULBP2	144
ULBP5	1.55
ULBP6	0.15

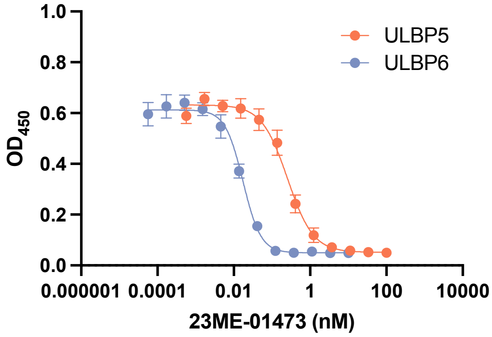

NKG2DL	IC50 (nM)
ULBP5	0.31
ULBP6	0.04

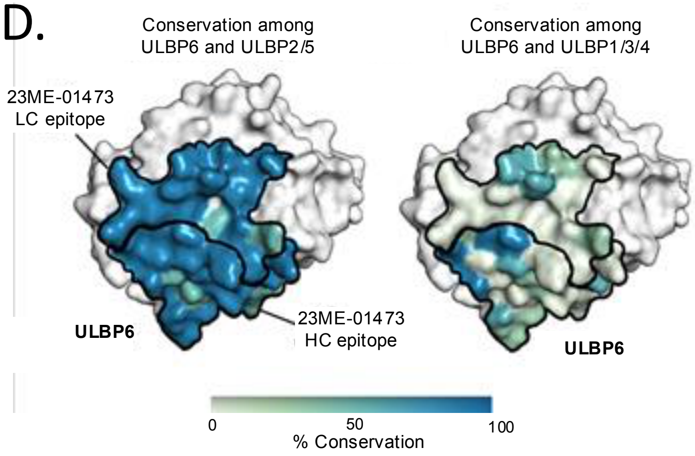

Supplementary Figure S6: Amino acid sequence alignment of human ULBPs and binding characterization of 23ME-01473

A) Jalview-derived alignment of the amino acid sequences of the extracellular domains of human ULBP1 (Uniprot ID Q9BZM6), ULBP2 (Uniprot ID Q9BZM5), ULBP3 (Uniprot ID Q9BZM4), ULBP4 (Uniprot ID Q8TD07), ULBP5 (Uniprot ID Q6H3X3), and ULBP6 (Uniprot ID Q5VY80, which has the same sequence identity as ULBP6-02). The position of the alignment is colored according to the degree of conservation across all ULBPs, with dark purple being highly conserved positions, light purple being moderately conserved, and white being not conserved. Amino acid variants unique to ULBP6-01 or shared between ULBP6-01 and ULBP6-02 are highlighted by a green or purple boxed amino acid, respectively. Glycosylation sites are labeled as "Glyc." **B)** Dose-response curves used to determine the EC_{80} of ULBP2, ULBP5, and ULBP6 binding to NKG2D from an ELISA. Data represent mean ± SD of three technical replicates. **C)** Representative dose-response curves used to determine the IC_{50} of 23ME-01473 blocking the binding of ULBP5 and ULBP6 to NKG2D using an ELISA. The NKG2DL concentration corresponding to the EC_{80} was used. Data represent mean ± SD of three technical replicates from one of four independent experiments. **D)** Conservation of the 23ME-01473 paratope, encircled by solid black lines, on ULBP6. The colors represent the % amino acid conservation between ULBP6, ULBP2, and ULBP5 (left panel) and between ULBP6, ULBP1, ULBP3, and ULBP4 (right panel).
