# Supplementary material for: 23ME-01473, an Fc Effector–Enhanced Anti-ULBP6/2/5 Antibody, Restores NK Cell–Mediated Antitumor Immunity through NKG2D and FcγRIIIa Activation
Source: Cancer Res Commun. 2025 Mar 21;5(3):477–96. doi: 10.1158/2767-9764.CRC-24-0478 (PMC11927390; doi:10.1158/2767-9764.CRC-24-0478)
Supplement: Supplementary Figure S7 [file crc-24-0478_supplementary_figure_s7_suppsf7.pdf]

Supplementary Figure S7

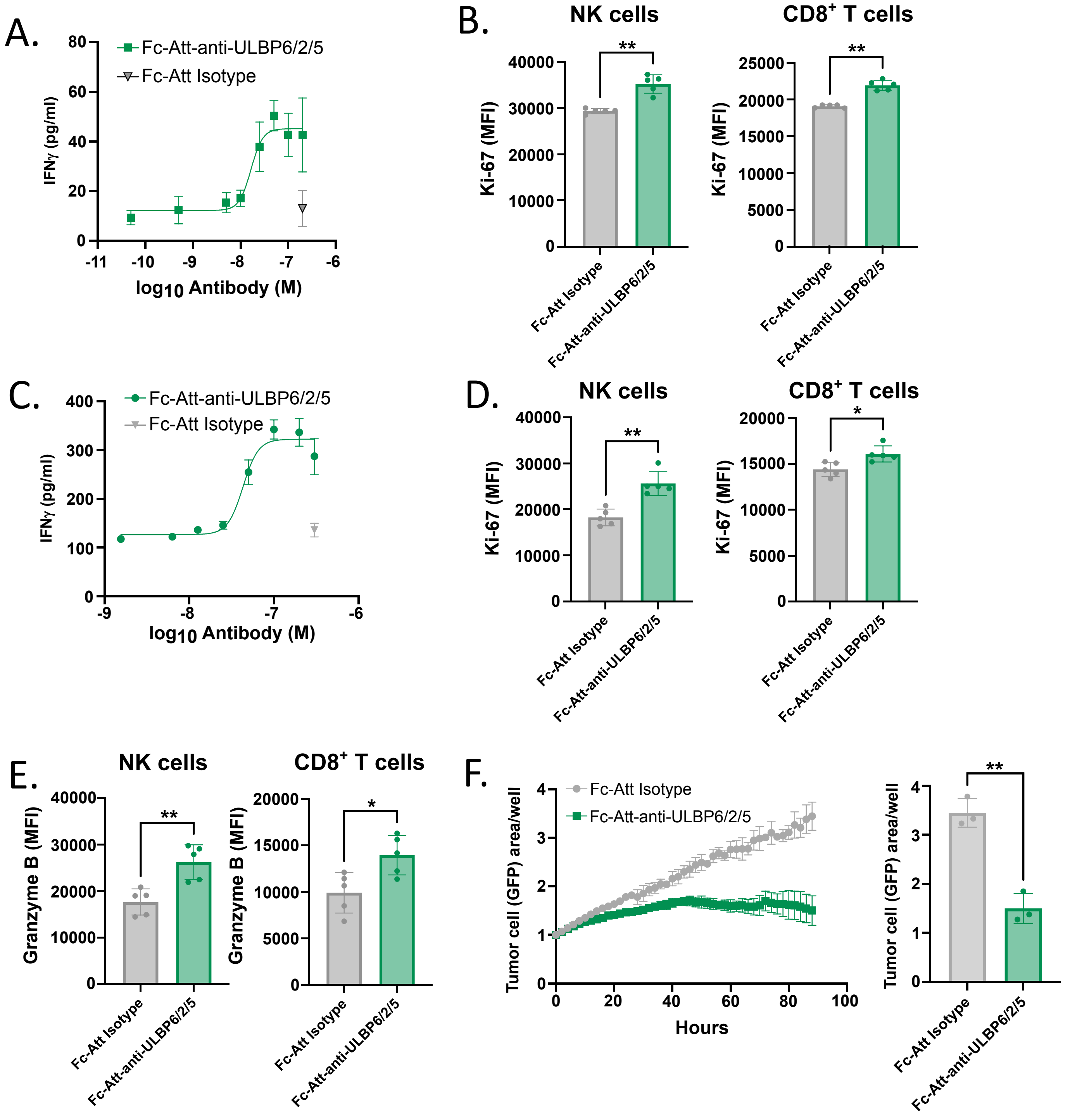

Supplementary Figure S7: Fc-Att-anti-ULBP6/2/5 increases IFNγ, granzyme B, and Ki-67 expression in immune cells

A) IFNγ concentration of the supernatants of IL–2/IL-15–primed PBMCs co-cultured with COV644 cells, 50 nM recombinant sULBP6-02, and 0.05 to 200 nM Fc-Att-anti-ULBP6/2/5 or 200 nM Fc-Att Isotype control. Data represent mean ± SD of three technical replicates per condition from one of three biological replicates. **B)** IL-2/IL-15-primed PBMCs were co-cultured with COV644 cells, 50 nM recombinant sULBP6-02, and 200 nM Fc-Att-anti-ULBP6/2/5 or Fc-Att isotype control for 48 hours. PBMCs were harvested for flow cytometric analysis of Ki-67 intracellular staining in NK and $CD8^+$ T cells. Data represent mean ± SD of five technical replicates per condition from one of two biological replicates. Mann-Whitney test was used for statistical analysis. **C)** IFNγ concentration of the supernatants of IL-2/IL-15-primed PBMCs cultured with 200 nM plate-bound MICA, 50 nM recombinant sULBP6-02, and 0.05 to 200 nM Fc-Att-anti-ULBP6/2/5 or 200 nM Fc-Att isotype control for 24 hours. Data represent mean ± SD of four technical replicates per condition from one of three biological replicates. PBMCs were cultured on 200 nM plate-bound MICA and 200 nM Fc-Att-anti-ULBP6/2/5 or Fc-Att isotype control for 48 hours, then harvested and analyzed for **D)** Ki-67 and **E)** granzyme B expression in NK and $CD8^+$ T cells. Data represent mean ± SD of five technical replicates per condition from one of two biological replicates. Mann-Whitney test was used for statistical analysis. **F)** Quantification of COV644-GFP cell growth, as measured by GFP area per well by an IncuCyte live cell analysis system, in the presence of NK cells, 50 nM recombinant sULBP6-02, and 100 nM Fc-Att-anti-ULBP6/2/5 or Fc-Att isotype control. Quantification is represented continuously over a 5 day time-course (left panel) and at the end of the 5 day timepoint (right panel). Data represent mean ± SD of three technical replicates per condition from one of three biological replicates. Unpaired t test was used for statistical analysis. * $P \leq 0.05$, ** $P \leq 0.01$.
