# Supplementary material for: 23ME-01473, an Fc Effector–Enhanced Anti-ULBP6/2/5 Antibody, Restores NK Cell–Mediated Antitumor Immunity through NKG2D and FcγRIIIa Activation
Source: Cancer Res Commun. 2025 Mar 21;5(3):477–96. doi: 10.1158/2767-9764.CRC-24-0478 (PMC11927390; doi:10.1158/2767-9764.CRC-24-0478)
Supplement: Supplementary Figure S8 [file crc-24-0478_supplementary_figure_s8_suppsf8.pdf]

# Supplementary Figure S8

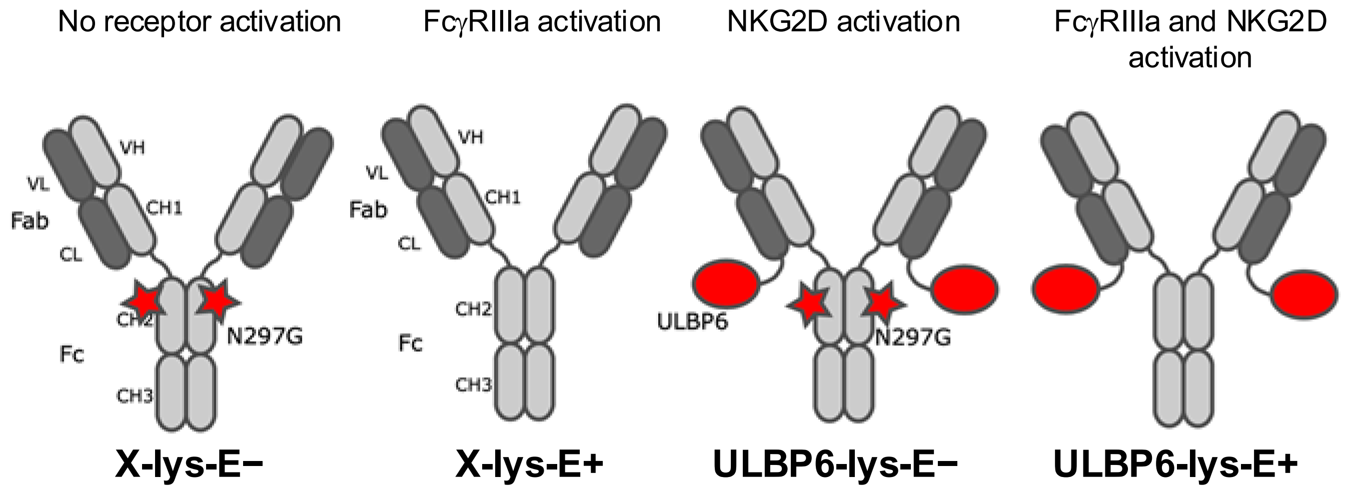


**Supplementary Figure S8: Activation of NKG2D and FcγRIIIa is synergistic**
Schematic of tool antibodies that stimulate neither NKG2D nor FcγRIIIa (Fc-Att-anti-lysozyme; X-lyse-E-), FcγRIIIa (Fc-WT-anti-lysozyme; X-lyse-E+), NKG2D (Fc-Att-anti-lysozyme with ULBP6 fused to the light chain; ULBP6-lyse-E-), or NKG2D and FcγRIIIa (Fc-WT-anti-lysozyme with ULBP6 fused to the light chain; ULBP6-lys-E+). Red stars represent the N297G mutation in the Fc domain, and red ovals represent ULBP6 that is fused to the light chain. Abbreviations: CH1-3=constant heavy chain domains 1-3, CL= constant light chain, Fab= fragment antigen-binding region, Fc=fragment crystallizable region, VH=variable heavy chain, VL=variable light chain.
