# Supplementary material for: 23ME-01473, an Fc Effector–Enhanced Anti-ULBP6/2/5 Antibody, Restores NK Cell–Mediated Antitumor Immunity through NKG2D and FcγRIIIa Activation
Source: Cancer Res Commun. 2025 Mar 21;5(3):477–96. doi: 10.1158/2767-9764.CRC-24-0478 (PMC11927390; doi:10.1158/2767-9764.CRC-24-0478)
Supplement: Supplementary Figure S9 [file crc-24-0478_supplementary_figure_s9_suppsf9.pdf]

Supplementary Figure S9

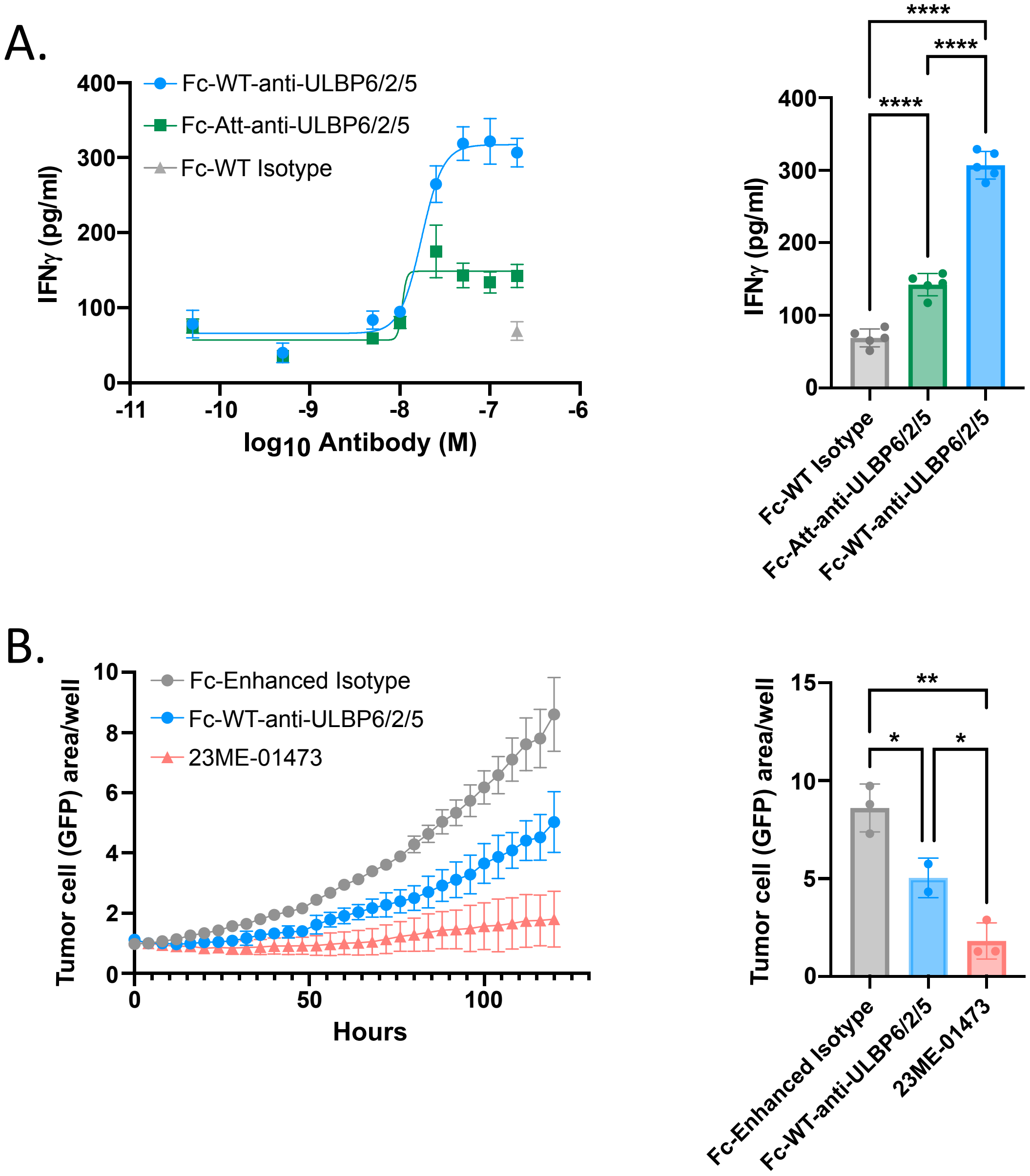

Supplementary Figure S9: Enhanced FcγRIIIa activation results in augmented PBMC-mediated IFNγ production and tumor cell killing

A) IFNγ concentration of the supernatants of IL-2/IL-15-primed PBMCs were co-cultured with COV644 cells, 50 nM recombinant sULBP6-02, and 0.05 to 200 nM Fc-WT- or Fc-Att-anti-ULBP6/2/5, or 200 nM Fc-WT isotype control for 24 hours. Results are depicted as a concentration-dependent response (left panel) or in response to 200 nM of each antibody (right panel). Data represent mean ± SD of five technical replicates per condition from one of three biological replicates.
B) Quantification of COV644-GFP cell growth, as measured by GFP area per well by an IncuCyte live cell analysis system, in the presence of IL-2/IL-15-primed PBMCs co-cultured with 50 nM recombinant sULBP6-02, and 100 nM 23ME-01473, Fc-WT-anti-ULBP6/2/5, or Fc-enhanced isotype control. Quantification is represented continuously over a 5 day time-course (left panel) and at the end of the 5 day timepoint (right panel). Data represent mean ± SD of three technical replicates per condition from one of four biological replicates. One-way ANOVA was used for statistical analysis. * $P \leq 0.05$, ** $P \leq 0.005$, **** $P \leq 0.0001$.
