# Supplementary material for: 23ME-01473, an Fc Effector–Enhanced Anti-ULBP6/2/5 Antibody, Restores NK Cell–Mediated Antitumor Immunity through NKG2D and FcγRIIIa Activation
Source: Cancer Res Commun. 2025 Mar 21;5(3):477–96. doi: 10.1158/2767-9764.CRC-24-0478 (PMC11927390; doi:10.1158/2767-9764.CRC-24-0478)
Supplement: Supplementary Figure S10 [file crc-24-0478_supplementary_figure_s10_suppsf10.pdf]

Supplementary Figure S10

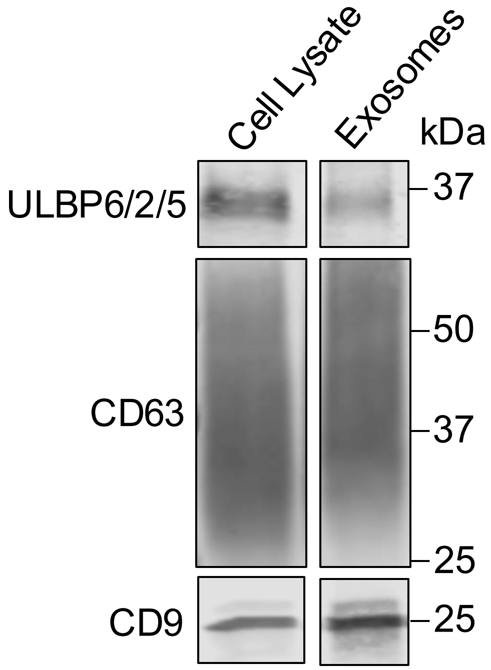

Supplementary Figure S10: COV644 cells produce ULBP6-expressing exosomes
COV644 cell lysates and exosomes isolated from COV644 cell supernatants were analyzed for ULBP6/2/5, CD63, and CD9 protein expression by Western blot. The same amount of total protein was loaded in each lane.
