# Supplementary material for: 23ME-01473, an Fc Effector–Enhanced Anti-ULBP6/2/5 Antibody, Restores NK Cell–Mediated Antitumor Immunity through NKG2D and FcγRIIIa Activation
Source: Cancer Res Commun. 2025 Mar 21;5(3):477–96. doi: 10.1158/2767-9764.CRC-24-0478 (PMC11927390; doi:10.1158/2767-9764.CRC-24-0478)
Supplement: Supplementary Data — List of collaborators from 23andMe Research Team [file crc-24-0478_supplementary_data_suppsdc.docx]

Collaborators, Affiliations

Stella Aslibekyan^2^

Adam Auton^2^

Robert K. Bell^2^

Katelyn Kukar Bond^2^

Zayn Cochinwala^2^

Sayantan Das^2^

Kahsaia de Brito^2^

Emily DelloRusso^2^

Chris Eijsbouts^2^

Sarah L. Elson^2^

Chris German^2^

Julie M. Granka^2^

Barry Hicks^2^

David A. Hinds^2^

Reza Jabal^2^

Aly Khan^2^

Matthew J. Kmiecik^2^

Alan Kwong^2^

Yanyu Liang^2^

Keng-Han Lin^2^

Matthew H. McIntyre^2^

Shubham Saini^2^

Anjali J. Shastri^2^

Jingchunzi Shi^2^

Suyash Shringarpure^2^

Qiaojuan Jane Su^2^

Vinh Tran^2^

Joyce Y. Tung^2^

Catherine H. Weldon^2^

Wanwan Xu^2^

^2^23andMe, Inc. Research, 223 N Mathilda Ave, Sunnyvale, CA 94086
